# Dual-anion regulation engineering enhances chloridion corrosion resistance for long-lasting industrial-scale seawater splitting

**DOI:** 10.1039/d5sc03775a

**Published:** 2025-07-29

**Authors:** Tianqi Gao, Wenzhe Wang, Zenong Zhang, Wanyu Li, Huanhuan Gao, Jiawei Liu, Xiaojun Zhao, Zhihong Liu, Yu Chen

**Affiliations:** a Key Laboratory for Macromolecular Science of Shaanxi Province, School of Chemistry and Chemical Engineering, Shaanxi Normal University Xi'an 710062 P. R. China liuzh@snnu.edu.cn; b Department of Chemical and Biological Engineering, The Hong Kong University of Science and Technology Clear Water Bay Kowloon Hong Kong 999077 P. R. China jiaweiliu@ust.hk; c School of Metallurgical Engineering, Xi'an University of Architecture and Technology Xi'an 710055 P. R. China xjzhao@xauat.edu.cn; d School of Materials Science and Engineering, Shaanxi Normal University Xi'an 710119 P. R. China chenyu001@snnu.edu.cn

## Abstract

Developing non-precious metal electrocatalysts with high activity and high chlorine (Cl^−^) corrosion resistance at industrial current densities remains challenging for large-scale seawater splitting. To address this problem, we rationally design an amorphous cobalt–iron layered double hydroxide with intercalated borate anions (B_4_O_5_(OH)_4_^2−^–CoFe-LDH) grown over crystalline sulfurized cobalt molybdate with a sulfate-rich surface (SO_4_^2−^–CoMoO_4_) nanohybrid (B_4_O_5_(OH)_4_^2−^–CoFe-LDH/SO_4_^2−^–CoMoO_4_). Through sulfidation and amorphous/crystalline interface construction, multiple synergistic effects are induced, effectively modulating the electronic structure, increasing the number of accessible active sites, and promoting electron transfer. The density functional theory calculations and *in situ* spectroscopy measurements demonstrate that the integration of B_4_O_5_(OH)_4_^2−^–CoFe-LDH and SO_4_^2−^–CoMoO_4_ synergistically optimizes the adsorption energy of intermediates, lowers the reaction energy barrier, and facilitates the formation of CoOOH active species, enhancing the catalytic activity for the oxygen evolution reaction. The unique B_4_O_5_(OH)_4_^2−^/SO_4_^2−^ dual-anion layers block the unfavorable adsorption of Cl^−^ and contribute to increased resistance to Cl^−^, enabling long-term corrosion protection for stable seawater splitting. Inspiringly, the B_4_O_5_(OH)_4_^2−^–CoFe-LDH/SO_4_^2−^–CoMoO_4_ nanohybrid stably sustains the industrial current density (1 A cm^−2^) in alkaline simulated seawater for 720 hours, with only a minimal concentration of hypochlorite (ClO^−^, 0.0003%) in the electrolyte.

## Abstract 2

The B_4_O_5_(OH)_4_^2−^–CoFe-LDH/SO_4_^2−^–CoMoO_4_ nanohybrid, with an amorphous/crystalline interface, enables efficient seawater splitting. Its dual B_4_O_5_(OH)_4_^2−^/SO_4_^2−^ layers suppress Cl^−^ corrosion, facilitating stable industrial-scale seawater splitting.

## Graphical abstract

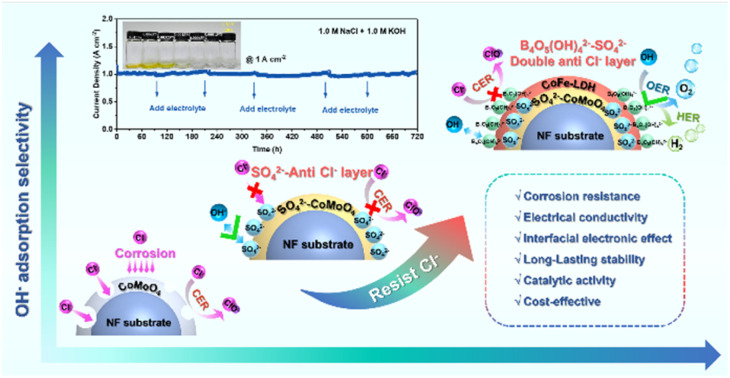

## Introduction

The escalating global demand for clean energy has spurred significant advancement in electrochemical water splitting, a key technology for producing green hydrogen (H_2_) with high energy density and zero carbon emissions.^1–3^ Currently, most electrochemical water splitting devices rely on freshwater as the feedstock, imposing a significant burden on limited global freshwater resources. In contrast, seawater, making up approximately 96.5% of Earth's water reserves, represents a more promising hydrogen reservoir.^4–6^ However, industrial-scale seawater splitting requires electrocatalysts capable of operating continuously for >100 hours at high current densities (≥200 mA cm^−2^) and elevated temperatures (60–90 °C), conditions that remain challenging for state-of-the-art electrocatalysts.^7,8^ A critical obstacle in seawater splitting is the anodic chlorine (Cl^−^) evolution reaction (CER), which competes with the oxygen evolution reaction (OER) through Cl^−^ oxidation and causes electrocatalyst corrosion *via* Cl^−^ adsorption.^9,10^ To overcome these challenges, the development of efficient electrocatalysts with optimized OER/CER selectivity and robust corrosion resistance is essential for enabling efficient and durable seawater splitting.

Two-dimensional (2D) layered double hydroxides (LDHs) are promising alternatives to commercial OER electrocatalysts such as IrO_2_ and RuO_2_,^11,12^ owing to their low cost, tunable composition, and high specific surface area.^13,14^ Additionally, LDHs also exhibit obvious activity toward the hydrogen evolution reaction (HER), enabling the utilization of LDH-based electrocatalysts as bifunctional water-splitting electrocatalysts. However, their practical applications face challenges, such as limited HER activity, aggregation of 2D nanosheets that reduces active site exposure, and insufficient long-term corrosion resistance in seawater splitting. To overcome these barriers, advanced electrocatalyst design strategies have been developed, such as anion intercalation, phase engineering, and interface engineering *via* introducing other high-activity materials.^15–17^ Recent studies have shown that the intercalated anions in LDHs can form a passivation layer to protect the electrocatalyst from corrosion by Cl^−^ in seawater.^18,19^ For example, the intercalation of carbonate into NiFe-LDH could reduce Cl^−^ adsorption on the electrocatalyst surface and hinder Cl^−^ corrosion.^20^ The facile insertion of WO_4_^2−^ into the interlayers of NiFe-LDH could influence the corrosion behavior during seawater oxidation significantly.^21^ Further improvements can be achieved through amorphous phase engineering.^22^ Amorphous LDHs feature disordered atomic arrangements, abundant unsaturated coordination sites, and defect-rich surfaces, which enhance active site exposure, facilitate reactant adsorption, and accelerate electron transfer,^23,24^ thereby boosting their electrocatalyst activity.^25^ For example, amorphous hollow CoNiFe-LDH nanocages have exhibited excellent electrocatalytic performance toward the OER due to their high density of active sites.^26^

Furthermore, constructing LDH-based nanomaterials with well-defined interfaces offers greater potential to enhance electrocatalytic performance through synergistic effects. The formation of hetero-interfaces enables electronic structure modulation *via* interfacial charge redistribution at the interface, mitigates LDH nanosheet stacking to maximize active site exposure, and induces the formation of defect sites and strain gradients, which collectively increase the active surface area and accelerate interfacial electron transfer. These effects optimize the adsorption energies of intermediates and improve reaction kinetics.^27–29^ For instance, a crystalline NiCoFeP core–amorphous NiCoFe-LDH shell nanohybrid exhibited excellent activity and stability as a result of the synergistic effect between the amorphous and crystalline phases.^30^ Among various LDH-based nanomaterials, the integration of cobalt molybdate (CoMoO_4_) stands out as a promising candidate due to its facile synthesis, robust structural stability, abundance of active sites created by the synergistic interaction between Co and Mo atoms, and its bifunctional capability for both HER and OER.^31–33^ More importantly, its adaptability for structural modifications, such as anion doping, enables tailored interface engineering to further refine electronic properties and electrocatalytic performance. Taken together, the integration of anion intercalation, amorphous phase engineering, and interface engineering in LDHs offers a three-pronged approach to simultaneously mitigate Cl^−^ corrosion and optimize intermediate adsorption energies, thereby boosting the electrocatalytic activity and durability for seawater splitting.

Inspired by the above insights, a nanohybrid of amorphous CoFe-LDH nanosheets with intercalated borate anions grown over crystalline CoMoO_4_ nanorods with a sulfate-rich surface (denoted as B_4_O_5_(OH)_4_^2−^–CoFe-LDH/SO_4_^2−^–CoMoO_4_) was constructed by combining the strategies of anion intercalation, phase engineering, and interface engineering. A combination of electrochemical studies, structural characterization of pre- and post-electrolysis electrocatalysts, *in situ* spectroscopy measurements, and density functional theory (DFT) studies together demonstrated that the formation of the amorphous/crystalline interface facilitated two-phase synergy, resulting in a modulated electronic structure, accelerated electron transfer, and abundant active sites. More importantly, the intercalated B_4_O_5_(OH)_4_^2−^ in LDHs and SO_4_^2−^ on the CoMoO_4_ surface together form a highly negatively charged B_4_O_5_(OH)_4_^2−^/SO_4_^2−^ dual-anion layer that impedes Cl^−^ corrosion through electrostatic repulsion, ensuring robust durability for continuous seawater splitting at industrial current densities. As a result, the B_4_O_5_(OH)_4_^2−^–CoFe-LDH/SO_4_^2−^–CoMoO_4_ nanohybrid exhibited outstanding activity for both the HER and OER with overpotentials of 85 mV and 134 mV in 1.0 M KOH solution at 10 mA cm^−2^, respectively. Meanwhile, the B_4_O_5_(OH)_4_^2−^–CoFe-LDH/SO_4_^2−^–CoMoO_4_ nanohybrid also exhibited high OER selectivity in seawater splitting, with overpotentials of 190 and 182 mV in 1.0 M KOH and 1.0 M KOH + seawater at 100 mA cm^−2^, respectively. Additionally, the B_4_O_5_(OH)_4_^2−^–CoFe-LDH/SO_4_^2−^–CoMoO_4_ nanohybrid could maintain stable electrolysis for 720 hours under alkaline seawater conditions at an industrial current density of 1 A cm^−2^. Using the B_4_O_5_(OH)_4_^2−^–CoFe-LDH/SO_4_^2−^–CoMoO_4_ nanohybrid as a bifunctional electrocatalyst, the constructed electrolyzer required only 1.40 V (1.0 M KOH) and 1.43 V (1.0 M KOH + seawater) decomposition voltage to achieve 10 mA cm^−2^ current density.

## Results and discussion

**Fig. 1:**
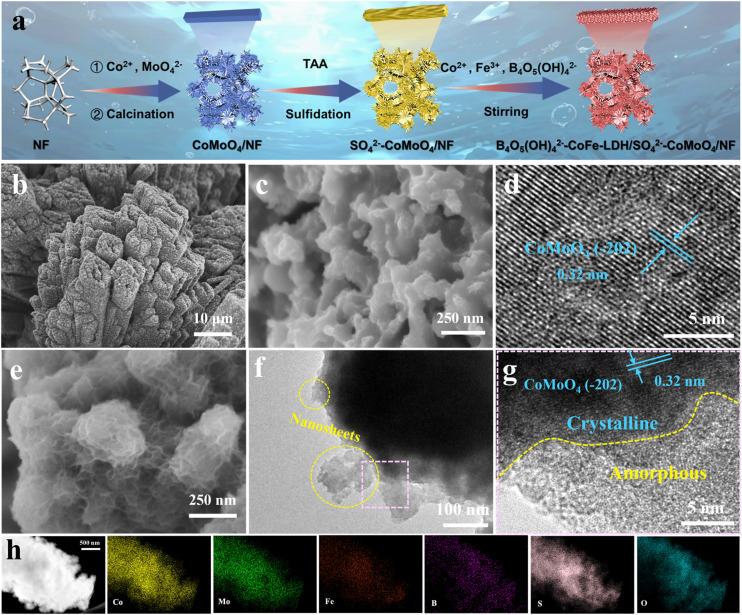
(a) Schematic synthetic procedures of the B_4_O_5_(OH)_4_^2−^–CoFe-LDH/SO_4_^2−^–CoMoO_4_ nanohybrid. (b and c) SEM and (d) HRTEM images of SO_4_^2−^–CoMoO_4_ nanorods. (e) SEM, (f) TEM, (g) HRTEM and (h) HAADF-STEM images and the corresponding EDX elemental maps of the B_4_O_5_(OH)_4_^2−^–CoFe-LDH/SO_4_^2−^–CoMoO_4_ nanohybrid.

Fig. 1a depicts the fabrication process of the B_4_O_5_(OH)_4_^2−^–CoFe-LDH/SO_4_^2−^–CoMoO_4_ nanohybrid *via* a three-step synthetic protocol. Firstly, CoMoO_4_ nanorods were uniformly grown on the nickel foam (NF) surface *via* a conventional hydrothermal method, followed by calcination to form a vertically aligned crystalline structure. Subsequently, the sulfidation-treated CoMoO_4_ nanorods were exposed to air, resulting in CoMoO_4_ with a surface rich in sulfate groups (SO_4_^2−^–CoMoO_4_). Sulfidation not only induces surface roughness, which facilitates the growth of LDHs, but also forms a surface SO_4_^2−^ passivation layer capable of electrostatically repelling Cl^−^ in seawater, thereby effectively mitigating the CER. Finally, amorphous B_4_O_5_(OH)_4_^2−^–CoFe-LDH was instantaneously deposited onto the SO_4_^2−^–CoMoO_4_ surface *via* a self-limiting reaction under ambient temperature stirring (25 °C), resulting in the formation of a hierarchical B_4_O_5_(OH)_4_^2−^–CoFe-LDH/SO_4_^2−^–CoMoO_4_ nanohybrid. The amorphous 2D B_4_O_5_(OH)_4_^2−^–CoFe-LDH nanosheets introduce abundant active sites, significantly boosting electrocatalytic efficiency. Moreover, the intercalation of B_4_O_5_(OH)_4_^2−^ into CoFe-LDH forms an additional passivation protective layer, which together with the SO_4_^2−^ anions on the CoMoO_4_ surface, constructs a B_4_O_5_(OH)_4_^2−^/SO_4_^2−^ dual-anion protective barrier, further enhancing resistance against Cl^−^ corrosion in seawater.

The morphology and structure of the B_4_O_5_(OH)_4_^2−^–CoFe-LDH/SO_4_^2−^–CoMoO_4_ nanohybrid were first examined by electron microscopy. As depicted in the scanning electron microscopy (SEM) pattern (Fig. S1), pure rod-like CoMoO_4_ crystals with smooth surfaces are vertically aligned on NF to form a self-supported robust three-dimensional (3D) hierarchical architecture. The corresponding elemental mapping reveals uniform distribution of Co, Mo, and O throughout the CoMoO_4_. After sulfidation, SO_4_^2−^–CoMoO_4_ still maintains its original rod-like structure (Fig. 1b). Meanwhile, the previously smooth surface becomes rough (Fig. 1c), accompanied by a uniform distribution of Co, Mo, O, and S (Fig. S2). The sulfidation duration is optimized to assess the effect of the degree of sulfidation on the electrocatalytic activity. As shown in Fig. S3, slight sulfidation is insufficient to change the unique and stable structure of CoMoO_4_ (SO_4_^2−^–CoMoO_4_-1 h, one-hour sulfidation). In contrast, excessive sulfidation compromises its robust framework (SO_4_^2−^–CoMoO_4_-6 h), causing the collapse of nanopillars and severely destabilizing the overall structure of CoMoO_4_ (Fig. S4). Upon evaluating the OER performance of CoMoO_4_-based electrocatalysts subjected to different sulfidation durations (Fig. S5), SO_4_^2−^–CoMoO_4_-3 h (three-hour sulfidation) was selected as the optimized electrocatalyst configuration for subsequent experiments. To streamline notation, SO_4_^2−^–CoMoO_4_-3 h is hereinafter denoted as SO_4_^2−^–CoMoO_4_ in this text. The high-resolution transmission electron microscopy (HRTEM) image clearly shows a lattice spacing of 0.32 nm, corresponding to the (−202) facet of CoMoO_4_ (Fig. 1d), indicating the preservation of the CoMoO_4_ crystal structure after sulfidation.^34^ Subsequently, the B_4_O_5_(OH)_4_^2−^–CoFe-LDH nanosheets were uniformly grown on the outer layer of SO_4_^2−^–CoMoO_4_ nanorods *via* a conventional *in situ* hydrolysis process, resulting in the formation of a hierarchical B_4_O_5_(OH)_4_^2−^–CoFe-LDH/SO_4_^2−^–CoMoO_4_ nanohybrid (Fig. 1e and S6). The thickness of nanosheet coating is another critical factor, as further investigation into the stirring duration revealed that prolonged stirring causes nanosheet stacking, hindering the exposure of active sites and consequently reduces electrocatalytic efficiency (Fig. S7). Upon evaluating the OER performance of SO_4_^2−^–CoMoO_4_ nanorods coated with B_4_O_5_(OH)_4_^2−^–CoFe-LDH of different thicknesses (Fig. S8), the stirring time was optimized to 3 h (denoted as B_4_O_5_(OH)_4_^2−^–CoFe-LDH/SO_4_^2−^–CoMoO_4_ in this text). The TEM image further confirms the attachment of B_4_O_5_(OH)_4_^2−^–CoFe-LDH nanosheets to SO_4_^2−^–CoMoO_4_ nanorods (Fig. 1f). The corresponding HRTEM image in Fig. 1g clearly shows an amorphous/crystalline interface, where the inner crystalline region of CoMoO_4_ exhibits a lattice fringe of 0.32 nm corresponding to the (−202) plane, while the outer layer consists of amorphous B_4_O_5_(OH)_4_^2−^–CoFe-LDH. Selected area electron diffraction (SAED) analysis further confirms this structural configuration (Fig. S9). The SAED pattern exhibits distinct polycrystalline diffraction rings indexed to the (−202), (421), and (−532) planes of CoMoO_4_. In contrast, the absence of sharp diffraction features indicates the amorphous character of B_4_O_5_(OH)_4_^2−^–CoFe-LDH. Collectively, these findings confirm the successful formation of the amorphous/crystalline B_4_O_5_(OH)_4_^2−^–CoFe-LDH/SO_4_^2−^–CoMoO_4_ nanohybrid. As a comparison, without sulfidation treatment, B_4_O_5_(OH)_4_^2−^–CoFe-LDH cannot adhere uniformly to the smooth CoMoO_4_ surface (Fig. S10), indicating that surface roughening is essential to provide anchoring sites. The high-angle annular dark-field scanning TEM (HAADF-STEM) image and energy dispersive X-ray spectroscopy (EDX) maps display a uniform distribution of Co, Mo, Fe, B, S, and O elements throughout the sample (Fig. 1h).

**Fig. 2:**
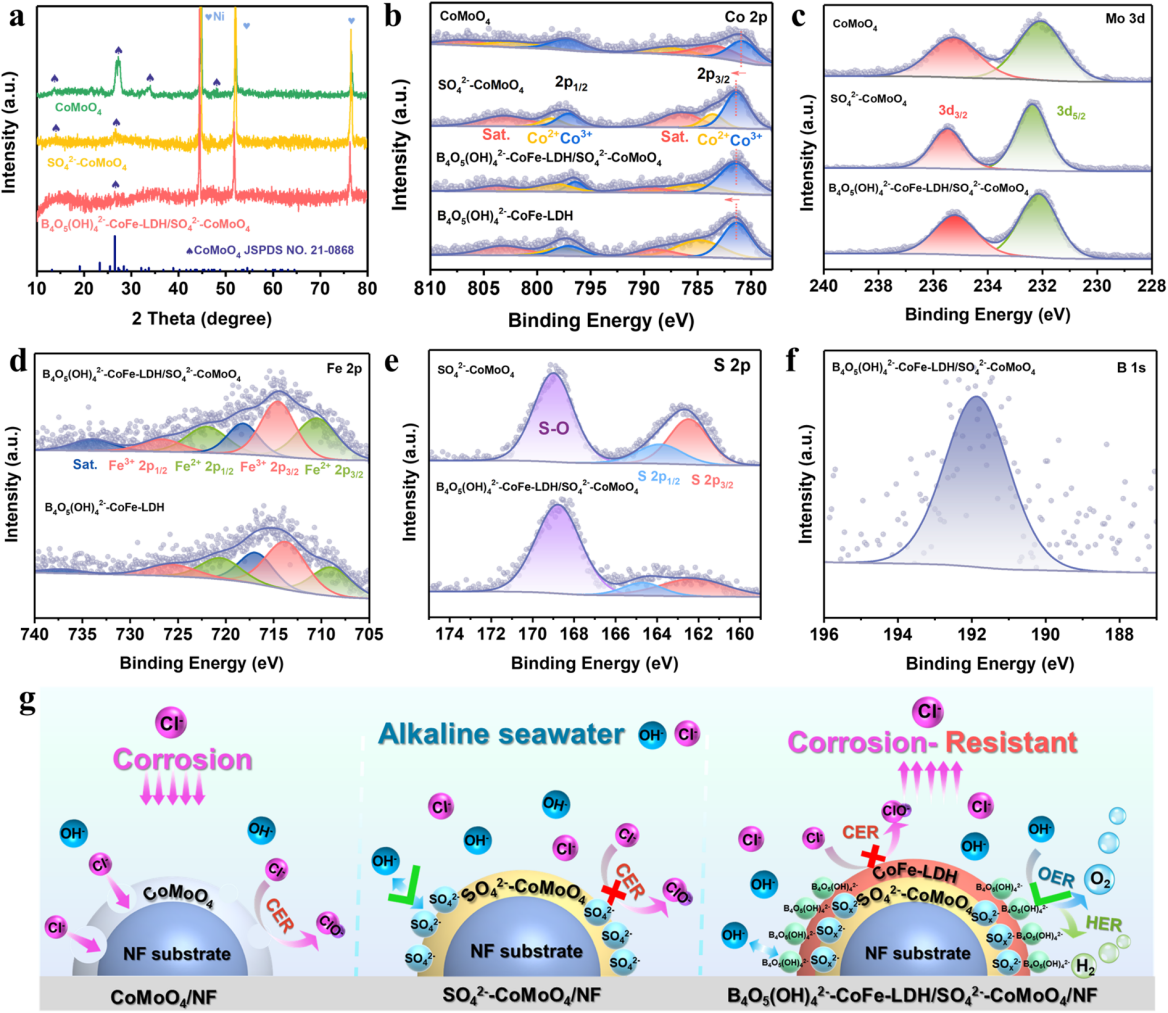
(a) XRD patterns of CoMoO_4_, SO_4_^2−^–CoMoO_4_, and B_4_O_5_(OH)_4_^2−^–CoFe-LDH/SO_4_^2−^–CoMoO_4_. (b) Co 2p, (c) Mo 3d, (d) Fe 2p, (e) S 2p, and (f) B 1s XPS spectra of B_4_O_5_(OH)_4_^2−^–CoFe-LDH/SO_4_^2−^–CoMoO_4_. (g) Schematic illustration of the dual corrosion-resistant layer induced by the surface SO_4_^2−^ and intercalated B_4_O_5_(OH)_4_^2−^ anions.

The crystal structure of the B_4_O_5_(OH)_4_^2−^–CoFe-LDH/SO_4_^2−^–CoMoO_4_ nanohybrid was characterized through X-ray diffraction (XRD) (Fig. 2a). The characteristic diffraction peaks of CoMoO_4_ at 13.6°, 27.3°, 33.9°, and 48.0° correspond to the (001), (−112), (−222) and (042) planes of CoMoO_4_ (PDF #21-0868). The SO_4_^2−^–CoMoO_4_ nanorods, retain the characteristic peaks associated with CoMoO_4_, although the peaks intensity is slightly diminished. After the growth of B_4_O_5_(OH)_4_^2−^–CoFe-LDH, no additional characteristic diffraction peaks are observed, indicating its amorphous nature. X-ray photoelectron spectroscopy (XPS) is performed to investigate the surface chemical composition and valence states of Co, Mo, and Fe. In the Co 2p spectrum of B_4_O_5_(OH)_4_^2−^–CoFe-LDH/SO_4_^2−^–CoMoO_4_, the peaks corresponding to Co^3+^ 2p_1/2_/Co^3+^ 2p_3/2_ are observed at 796.4/781.3 eV, while those for Co^2+^ 2p_1/2_/Co^2+^ 2p_3/2_ appear at 798.4/785.3 eV, accompanied by two satellite peaks at 790.1/803.8 eV (Fig. 2b).^35^ Compared to pure CoMoO_4_, the Co 2p binding energy in SO_4_^2−^–CoMoO_4_ exhibits a positive shift of 0.6 eV, indicating that replacing O with the less electronegative S reduces local electron affinity, leading to electron transfer from the Co environment to S to maintain charge balance. This shift confirms the electron transfer in SO_4_^2−^–CoMoO_4_, demonstrating that sulfidation alters the electronic structure of CoMoO_4_. Upon the overgrowth of B_4_O_5_(OH)_4_^2−^–CoFe-LDH, the overall content of Co^3+^ increases from 50% to 63%, suggesting that the incorporation of Fe^3+^ facilitates the formation of active species Co^3+^.^36^ In the Mo 3d spectrum of B_4_O_5_(OH)_4_^2−^–CoFe-LDH/SO_4_^2−^–CoMoO_4_, two prominent peaks at 232.1 and 235.2 eV are observed, corresponding to the Mo^6+^ species, indicating that Mo atoms are in a high oxidation state (Fig. 2c). Notably, sulfur doping induces a 0.3 eV shift of the CoMoO_4_ peak toward higher binding energy, resembling the migration trend of the Co element, further demonstrating that sulfidation can alter the electronic structure of the electrocatalyst.

In the Fe 2p spectrum of B_4_O_5_(OH)_4_^2−^–CoFe-LDH/SO_4_^2−^–CoMoO_4_ (Fig. 2d), two distinct peaks at 710.4 and 721.9 eV are assigned to Fe^2+^ species (2p_3/2_ and 2p_1/2_), while additional peaks observed at 714.5 and 726.6 eV correspond to the higher-valence Fe^3+^ species.^37–39^ Compared to B_4_O_5_(OH)_4_^2−^–CoFe-LDH, the Fe 2p_3/2_ peak in B_4_O_5_(OH)_4_^2−^–CoFe-LDH/SO_4_^2−^–CoMoO_4_ shifts to higher binding energy by 1.4 eV, while the content of Co^3+^ increases from 50% to 63%. Obviously, this change implies that Fe actively participates in surface charge redistribution, likely donating electrons to adjacent Co centers, thereby facilitating the formation of Co^3+^ species. This electron migration across the B_4_O_5_(OH)_4_^2−^–CoFe-LDH/SO_4_^2−^–CoMoO_4_ interface highlights the strong interaction between B_4_O_5_(OH)_4_^2−^–CoFe-LDH and SO_4_^2−^–CoMoO_4_. In the S 2p spectrum of B_4_O_5_(OH)_4_^2−^–CoFe-LDH/SO_4_^2−^–CoMoO_4_, the peaks at 164.7 and 162.2 eV correspond to S 2p_1/2_ and S 2p_3/2_, respectively, indicating that sulfur partially substitutes oxygen to form metal–sulfur bonds (Fig. 2e).^40,41^ Additionally, the peak at 168.8 eV corresponds to S–O bonds due to surface oxidation, suggesting the presence of surface-rich SO_4_^2−^,^26^ which aligns with the results of Fourier transform infrared spectroscopy (FTIR) (Fig. S11). The absorption peak at 1085 cm^−1^ is attributed to the asymmetric stretching vibration of S=O bonds.^42^ Further analysis using Raman spectroscopy reveals a peak at 1000 cm^−1^, which can be attributed to S–O bonds (Fig. S12),^43^ providing evidence for the formation of abundant SO_4_^2−^ anions on the surface. In the B 1s spectrum, the peak at 191.8 eV is assigned to the B–O bond in borate anions (Fig. 2f), which is consistent with the results of the FTIR spectrum (Fig. S11). Specifically, the absorption peaks at 1024 and 814 cm^−1^ originate from the asymmetric and symmetric stretching of B(4)–O, respectively, while the peak at 1278 cm^−1^ corresponds to the in-plane bending vibration of B–O–H within the BO_4_ group. The absorption peaks at 814 and 1355 cm^−1^ are assigned to the symmetric and asymmetric stretching of B(3)–O, and the peak at 691 cm^−1^ represents the out-of-plane bending of B(3)–O.^44^ All these assignments indicate that the intercalated borate exists in the form of B_4_O_5_(OH)_4_^2−^. Overall, sulfidation and the construction of an amorphous crystalline interface significantly induce electronic interaction between the two components, thereby modulating the electronic structure of active sites and influencing the adsorption energies of intermediates. More importantly, the formation of the B_4_O_5_(OH)_4_^2−^/SO_4_^2−^ dual-anion anticorrosion barrier can electrostatically repel Cl^−^ in seawater, providing a solid foundation for seawater splitting (Fig. 2g).

**Fig. 3:**
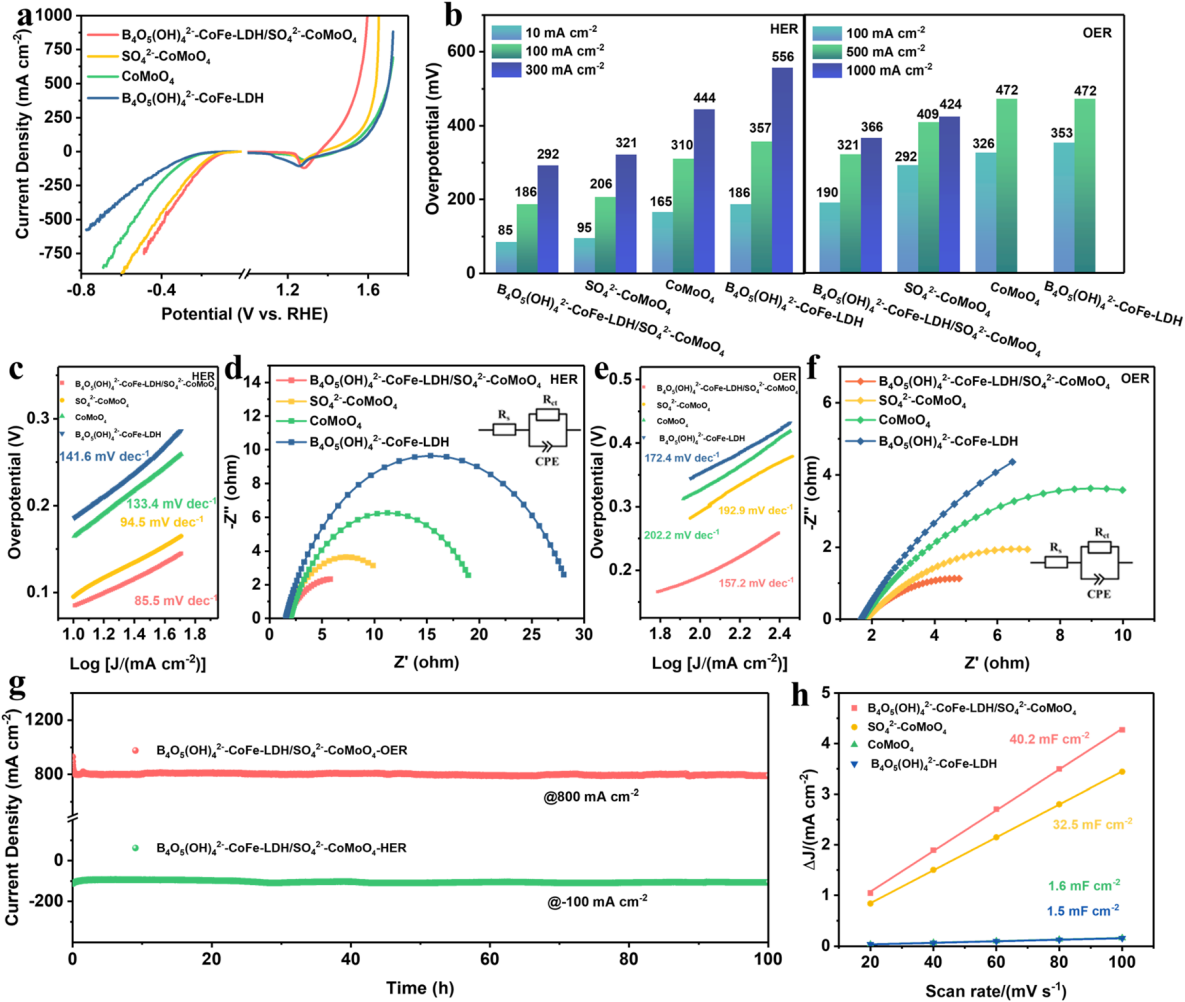
(a) LSV curves for the HER and OER in 1.0 M KOH solution. (b) Overpotentials at different current densities in the HER and OER. (c) Tafel slopes and (d) Nyquist plots (inset: the equivalent circuit) for the HER. (e) Tafel slopes and (f) Nyquist plots (inset: the equivalent circuit) for the OER. (g) *i*–*t* curves over 100 h for the OER and HER in 1.0 M KOH solution, respectively. (h) The *C*_dl_ of various samples in 1.0 M KOH solution.

Developing non-precious metal electrocatalysts that synergistically optimize both HER and OER performance remains a critical challenge for overall water splitting. The HER and OER activities of the B_4_O_5_(OH)_4_^2−^–CoFe-LDH/SO_4_^2−^–CoMoO_4_ nanohybrid were evaluated in 1.0 M KOH electrolyte (25 °C). As shown in Fig. 3a and b, the B_4_O_5_(OH)_4_^2−^–CoFe-LDH/SO_4_^2−^–CoMoO_4_ nanohybrid exhibits superior HER activity, with the lowest overpotentials of 85 and 186 mV at current densities of 10 and 100 mA cm^−2^, respectively, outperforming SO_4_^2−^–CoMoO_4_ (95 and 206 mV), CoMoO_4_ (165 and 310 mV), and B_4_O_5_(OH)_4_^2−^–CoFe-LDH (186 and 357 mV). These results suggest that the excellent HER activity of the B_4_O_5_(OH)_4_^2−^–CoFe-LDH/SO_4_^2−^–CoMoO_4_ nanohybrid may be attributed to the combined effects of sulfidation and crystalline/amorphous interface construction, which together provide a large surface area with plentiful active sites and a modulated electronic structure. Tafel plots were calculated to investigate the intrinsic kinetics of HER at different electrocatalysts. Tafel plots in Fig. 3c reveal that B_4_O_5_(OH)_4_^2−^–CoFe-LDH/SO_4_^2−^–CoMoO_4_ exhibits the fastest HER kinetics with a slope of 85.5 mV dec^−1^, significantly lower than those of SO_4_^2−^–CoMoO_4_ (94.5 mV dec^−1^), CoMoO_4_ (133.4 mV dec^−1^), and B_4_O_5_(OH)_4_^2−^–CoFe-LDH (141.6 mV dec^−1^). This result confirms the optimized Volmer–Heyrovsky reaction pathway,^45^ as the reduced slope suggests faster charge transfer and hydrogen desorption. Electrochemical impedance spectroscopy (EIS) was used to evaluate the electron transfer kinetics of the HER at different electrocatalysts. B_4_O_5_(OH)_4_^2−^–CoFe-LDH/SO_4_^2−^–CoMoO_4_ exhibits the smallest charge transfer resistance (*R*_ct_) in the HER process (Fig. 3d), while the other electrocatalysts follow the trend SO_4_^2−^–CoMoO_4_ < CoMoO_4_ < B_4_O_5_(OH)_4_^2−^–CoFe-LDH, confirming that sulfidation and the construction of the crystalline/amorphous interface facilitate rapid electron transfer and enhance HER activity.

Notably, the B_4_O_5_(OH)_4_^2−^–CoFe-LDH/SO_4_^2−^–CoMoO_4_ nanohybrid also exhibits superior OER activity. The B_4_O_5_(OH)_4_^2−^–CoFe-LDH/SO_4_^2−^–CoMoO_4_ nanohybrid delivers the lowest overpotential of 190 mV at 100 mA cm^−2^, outperforming SO_4_^2−^–CoMoO_4_ (292 mV), CoMoO_4_ (326 mV), and B_4_O_5_(OH)_4_^2−^–CoFe-LDH (353 mV). Even at an industrial-level current density of 1 A cm^−2^, the B_4_O_5_(OH)_4_^2−^–CoFe-LDH/SO_4_^2−^–CoMoO_4_ nanohybrid also exhibits a low overpotential of only 366 mV (Fig. 3a and b). Furthermore, the B_4_O_5_(OH)_4_^2−^–CoFe-LDH/SO_4_^2−^–CoMoO_4_ nanohybrid displays the smallest Tafel slope of 157.2 mV dec^−1^, in comparison with SO_4_^2−^–CoMoO_4_ (192.9 mV dec^−1^), CoMoO_4_ (202.2 mV dec^−1^), and B_4_O_5_(OH)_4_^2−^–CoFe-LDH (172.4 mV dec^−1^), indicating the fastest OER kinetics of the B_4_O_5_(OH)_4_^2−^–CoFe-LDH/SO_4_^2−^–CoMoO_4_ nanohybrid (Fig. 3e). The EIS curves in Fig. 3f indicate that the B_4_O_5_(OH)_4_^2−^–CoFe-LDH/SO_4_^2−^–CoMoO_4_ nanohybrid also exhibits the lowest *R*_ct_ during the OER, suggesting enhanced electron mobility efficiency. Collectively, the LSV, Tafel, and EIS results demonstrate that the B_4_O_5_(OH)_4_^2−^–CoFe-LDH/SO_4_^2−^–CoMoO_4_ nanohybrid exhibits superior HER and OER performance. The stability of the B_4_O_5_(OH)_4_^2−^–CoFe-LDH/SO_4_^2−^–CoMoO_4_ nanohybrid in 1.0 M KOH was evaluated. As shown in Fig. 3g, the B_4_O_5_(OH)_4_^2−^–CoFe-LDH/SO_4_^2−^–CoMoO_4_ nanohybrid maintains stable operation for nearly 100 hours, exhibiting excellent OER/HER stability. The morphology of B_4_O_5_(OH)_4_^2−^–CoFe-LDH/SO_4_^2−^–CoMoO_4_ remains well preserved after the OER/HER LSV tests. As observed, B_4_O_5_(OH)_4_^2−^–CoFe-LDH nanosheets are clearly visible on the surface of robust SO_4_^2−^–CoMoO_4_ nanorods, further illustrating the structural stability of the electrocatalyst (Fig. S13 and S14).

The electrical properties of the B_4_O_5_(OH)_4_^2−^–CoFe-LDH/SO_4_^2−^–CoMoO_4_ nanohybrid were further characterized and analyzed. First, cyclic voltammetry tests were conducted in the non-faradaic region to determine the double-layer capacitance (*C*_dl_) and electrochemically active surface area (ECSA) of the electrocatalysts (Fig. 3h and S15). The B_4_O_5_(OH)_4_^2−^–CoFe-LDH/SO_4_^2−^–CoMoO_4_ nanohybrid exhibited the largest *C*_dl_ value of 40.2 mF cm^−2^, which is approximately 25 times greater than those of CoMoO_4_ (1.6 mF cm^−2^) and B_4_O_5_(OH)_4_^2−^–CoFe-LDH (1.5 mF cm^−2^), indicating that the B_4_O_5_(OH)_4_^2−^–CoFe-LDH/SO_4_^2−^–CoMoO_4_ nanohybrid possesses a significantly enlarged ECSA and more accessible active sites. Since surface wettability is a critical interfacial chemical parameter for determining the overall electrocatalytic performance, the surface wettability of the electrocatalysts was characterized by water contact angle measurements. In Fig. S16, the contact angle of SO_4_^2−^–CoMoO_4_ powder is measured to be 49.9°, whereas the B_4_O_5_(OH)_4_^2−^–CoFe-LDH/SO_4_^2−^–CoMoO_4_ powder exhibits a contact angle of 38.8°, indicating that the coating of highly hydrophilic B_4_O_5_(OH)_4_^2−^–CoFe-LDH (water contact angle of 28.0°) on the SO_4_^2−^–CoMoO_4_ surface can enhance the hydrophilicity of the electrocatalyst. The increased hydrophilicity of the B_4_O_5_(OH)_4_^2−^–CoFe-LDH/SO_4_^2−^–CoMoO_4_ nanohybrid promotes the adsorption and penetration of the electrolyte, maximizing the ECSA and improving reaction kinetics.^46^

Besides, the N_2_ adsorption–desorption isotherms demonstrate that the B_4_O_5_(OH)_4_^2−^–CoFe-LDH/SO_4_^2−^–CoMoO_4_ nanohybrid (144.76 m^2^ g^−1^) has a much larger specific surface area than SO_4_^2−^–CoMoO_4_ (5.07 m^2^ g^−1^) and CoMoO_4_ (1.26 m^2^ g^−1^), which is one of the reasons why it has a large ECSA and high catalytic activity (Fig. S17). Moreover, the high electrocatalytic activity of the B_4_O_5_(OH)_4_^2−^–CoFe-LDH/SO_4_^2−^–CoMoO_4_ nanohybrid can also be ascribed to the plentiful active sites offered by the amorphous B_4_O_5_(OH)_4_^2−^–CoFe-LDH.^47^ To clarify this, the crystalline/crystalline B_4_O_5_(OH)_4_^2−^–CoFe-LDH/SO_4_^2−^–CoMoO_4_ nanohybrid was employed as a reference (Fig. S18). For the OER, the amorphous/crystalline B_4_O_5_(OH)_4_^2−^–CoFe-LDH/SO_4_^2−^–CoMoO_4_ nanohybrid exhibits a 60 mV lower overpotential than its crystalline/crystalline counterpart at 100 mA cm^−2^ (Fig. S19a). The crystalline/crystalline B_4_O_5_(OH)_4_^2−^–CoFe-LDH/SO_4_^2−^–CoMoO_4_ nanohybrid exhibits a larger Tafel slope (170.8 mV dec^−1^) than its amorphous/crystalline counterpart (157.2 mV dec^−1^), indicating slower reaction kinetics (Fig. S19 b). Similarly, the amorphous/crystalline B_4_O_5_(OH)_4_^2−^–CoFe-LDH/SO_4_^2−^–CoMoO_4_ nanohybrid achieves a 61 mV lower overpotential at 10 mA cm^−2^ and a reduced Tafel slope of 85.5 mV dec^−1^ compared to 132.7 mV dec^−1^ for the HER, further confirming that the amorphous/crystalline interface induces faster OER kinetics and enhanced charge transfer characteristics (Fig. S19c and d). This result demonstrates the significant advantages of constructing amorphous/crystalline structures for boosting OER catalytic efficiency. Capitalizing on these merits, the amorphous/crystalline B_4_O_5_(OH)_4_^2−^–CoFe-LDH/SO_4_^2−^–CoMoO_4_ nanohybrid exhibits superior OER/HER activity compared to the most recently reported transition metal electrocatalysts (Tables S1 and S2).

**Fig. 4:**
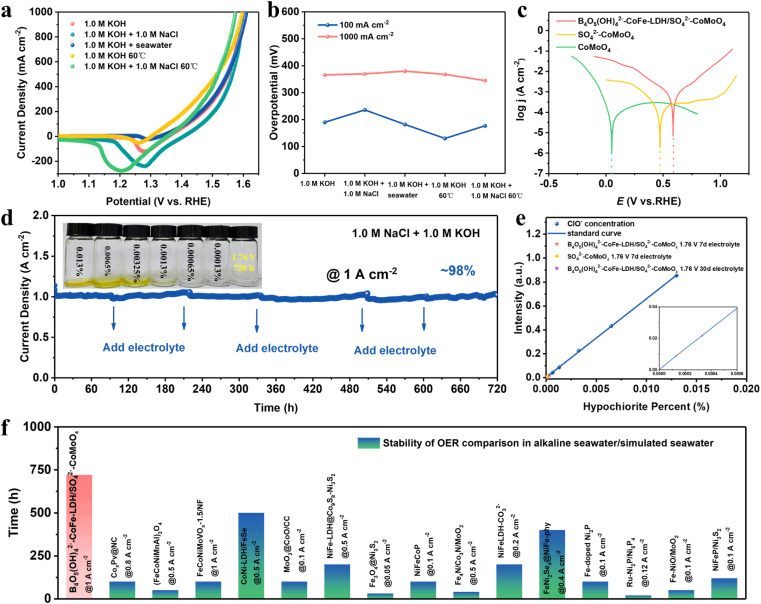
(a) LSV curves and (b) corresponding overpotentials for OER in different electrolytes. (c) The corrosion polarization curves in 1.0 M NaCl + 1.0 M KOH solution at different current densities. (d) *i*–*t* curve for OER in 1.0 M NaCl + 1.0 M KOH solution. (e) ClO^−^ concentration in the electrolyte after stability testing. (f) Comparison of the OER stability with other electrocatalysts reported in alkaline seawater/simulated seawater.

Building on the low overpotential and exceptional stability of the B_4_O_5_(OH)_4_^2−^–CoFe-LDH/SO_4_^2−^–CoMoO_4_ nanohybrid at high current densities in alkaline electrolytes (1.0 M KOH), its OER activity was further assessed in natural alkaline seawater (seawater + 1.0 M KOH) and simulated alkaline seawater (1.0 M NaCl + 1.0 M KOH) under the same conditions (Fig. 4a). Surprisingly, the B_4_O_5_(OH)_4_^2−^–CoFe-LDH/SO_4_^2−^–CoMoO_4_ nanohybrid reveals enhanced electrocatalytic performance at the industrial temperature of 60 °C. This fact not only demonstrates its ability to withstand high temperature but also reveals that the high-temperature environment accelerates reaction kinetics.^48^ The LSV results indicate that the B_4_O_5_(OH)_4_^2−^–CoFe-LDH/SO_4_^2−^–CoMoO_4_ nanohybrid is well-suited for industrial electrolysis applications. At an industrial-level current density of 1 A cm^−2^, B_4_O_5_(OH)_4_^2−^–CoFe-LDH/SO_4_^2−^–CoMoO_4_ can achieve remarkably low overpotentials of 370 and 380 mV in both simulated alkaline seawater and natural alkaline seawater, respectively, only 4 and 14 mV higher than those in 1.0 M KOH (366 mV). In contrast, B_4_O_5_(OH)_4_^2−^–CoFe-LDH, SO_4_^2−^–CoMoO_4_, and CoMoO_4_ exhibit significantly larger overpotential increases of 40, 44, and 75 mV in seawater environments, respectively (Fig. S20). This demonstrates that the B_4_O_5_(OH)_4_^2−^/SO_4_^2−^ dual-anion protective layer effectively mitigates Cl^−^-induced corrosion. Across different electrolytes and temperatures, the B_4_O_5_(OH)_4_^2−^–CoFe-LDH/SO_4_^2−^–CoMoO_4_ nanohybrid exhibits similar electrocatalytic activity, especially at 1 A cm^−2^; the overpotential shows relatively small fluctuation at the industrially relevant temperature of 60 °C (Fig. 4b). In addition, the B_4_O_5_(OH)_4_^2−^–CoFe-LDH/SO_4_^2−^–CoMoO_4_ nanohybrid maintains stable electrolysis at 800 mA cm^−2^ in simulated alkaline seawater for 120 hours at 60 °C (Fig. S21), further demonstrating its excellent durability under industrial conditions. This exceptional OER performance in seawater underscores the electrocatalyst's superior resistance to Cl^−^ corrosion. Fig. 4c illustrates the corrosion resistance of different electrocatalysts. In the 1.0 M NaCl + 1.0 M KOH electrolyte, the corrosion potential of B_4_O_5_(OH)_4_^2−^–CoFe-LDH/SO_4_^2−^–CoMoO_4_, SO_4_^2−^–CoMoO_4_, and CoMoO_4_ decreases sequentially, indicating an enhanced corrosion resistance in this order. This observation highlights the dual-anion protective mechanism of B_4_O_5_(OH)_4_^2−^/SO_4_^2−^ species in suppressing Cl^−^-induced corrosion *via* electrostatic repulsion. Specifically, the B_4_O_5_(OH)_4_^2−^ anion in CoFe-LDH plays a pivotal role in enhancing electrocatalyst's corrosion resistance by forming the first passivation layer. Meanwhile, the SO_4_^2−^ adsorbed on the CoMoO_4_ nanorod surface contributes to the formation of the second passivation layer, providing additional protection. Together, the dual B_4_O_5_(OH)_4_^2−^/SO_4_^2−^ layers facilitate efficient seawater splitting under harsh chloride-rich conditions.

As is commonly known, the CER triggered by abundant Cl^−^ in seawater can degrade OER activity and compromise the long-term stability of electrocatalysts, posing a significant challenge for direct seawater electrolysis for H_2_ production. Fig. 4d shows that the B_4_O_5_(OH)_4_^2−^–CoFe-LDH/SO_4_^2−^–CoMoO_4_ nanohybrid has exceptional stability and resistance to Cl^−^ corrosion, maintaining ∼98% of its initial current density after continuous electrolysis for 720 hours at 1 A cm^−2^ (at 1.76 V *vs.* RHE, exceeding the theoretical potential of 1.72 V *vs.* RHE for the competing CER process) for 720 hours.^49,50^ After 720 h of electrolysis, SEM and elemental mapping images reveal a well-preserved nanostructure and uniform distribution of Co, Mo, Fe, S, O, and B (Fig. S22), confirming excellent morphological stability. The prolonged and stable OER performance indicates that the B_4_O_5_(OH)_4_^2−^–CoFe-LDH/SO_4_^2−^–CoMoO_4_ nanohybrid possesses a stronger selective adsorption capacity for OH^−^ over Cl^−^ in seawater. To further validate the OH^−^ adsorption capability of the electrocatalyst, the concentration of hypochlorite (ClO^−^, the product of the CER) is analyzed. The ClO^−^ concentration gradient is established by measuring the absorbance of *o*-toluidine-NaClO solutions using UV-visible spectroscopy (Fig. S23). The post-test electrolyte ClO^−^ concentration is measured after 720 h of continuous electrolysis at 1.76 V (Fig. 4e). The negligible ClO^−^ accumulation (<0.0003% solution concentration) confirms effective OH^−^ adsorption and suppression of competitive Cl^−^ oxidation. Furthermore, the ClO^−^ concentrations in the electrolytes of SO_4_^2−^–CoMoO_4_ and B_4_O_5_(OH)_4_^2−^–CoFe-LDH/SO_4_^2−^–CoMoO_4_ were also measured after 168 h of electrolysis at 1.76 V. The SO_4_^2−^–CoMoO_4_ exhibited a ClO^−^ concentration nearly five times higher than that of B_4_O_5_(OH)_4_^2−^–CoFe-LDH/SO_4_^2−^–CoMoO_4_, further demonstrating that the integration of the B_4_O_5_(OH)_4_^2−^–CoFe-LDH layer plays a critical role in resisting Cl^−^ corrosion and enhancing OH^−^ selectivity. The excellent Cl^−^ corrosion resistance of the B_4_O_5_(OH)_4_^2−^–CoFe-LDH/SO_4_^2−^–CoMoO_4_ nanohybrid originates from a dual-anion protection mechanism. The intercalated B_4_O_5_(OH)_4_^2−^ within the CoFe-LDH layer provides steric hindrance and strong OH^−^ selectivity *via* large ionic radius and hydrogen bonding, effectively blocking Cl^−^ intrusion and stabilizing the layered structure. Meanwhile, surface-anchored SO_4_^2−^ on CoMoO_4_ forms an electrostatic repulsion layer that prevents Cl^−^ adsorption and preserves the structural integrity of the oxide phase during long-term electrolysis. Thanks to the excellent corrosion resistance, the B_4_O_5_(OH)_4_^2−^–CoFe-LDH/SO_4_^2−^–CoMoO_4_ nanohybrid demonstrates seawater splitting stability surpassing that of most recently reported electrocatalysts (Fig. 4f).^8,9,15,20,51–61^

**Fig. 5:**
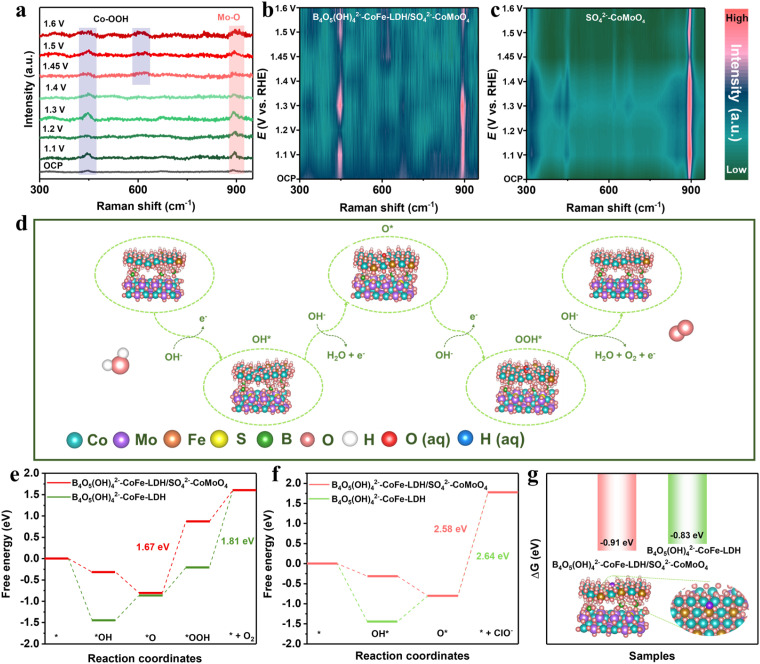
(a) *In situ* Raman spectra of B_4_O_5_(OH)_4_^2−^–CoFe-LDH/SO_4_^2−^–CoMoO_4_ for the OER in 1.0 M KOH solution under varying potentials, and (b and c) corresponding contour plots of B_4_O_5_(OH)_4_^2−^–CoFe-LDH/SO_4_^2−^–CoMoO_4_ and SO_4_^2−^–CoMoO_4_. (d) Reaction pathway diagram of the OER. (e) Gibbs free energy change of the OER. (f) Gibbs free energy change of the CER and (g) difference in Gibbs free energy change between the RDS of the OER and CER.

The performance enhancement mechanism of the B_4_O_5_(OH)_4_^2−^–CoFe-LDH/SO_4_^2−^–CoMoO_4_ nanohybrid was investigated *via in situ* Raman, *in situ* attenuated total reflection infrared spectroscopy (ATR-IR), and *ex situ* XPS. First, *in situ* Raman measurements were performed to probe the real-time surface reconstruction of the electrocatalyst during the OER process. As shown in Fig. 5a and b, two prominent Raman signals at 450 (E_g_ mode, O–Co–O bending) and 618 (A_1g_ mode, O–Co–O stretching) cm^−1^ are detected as the potential shifts positively, corresponding to the formation of CoOOH species.^62^ As the applied voltage increases to 1.45 V, the peak intensity at 618 cm^−1^ increases significantly, indicating the formation of the active species CoOOH during the surface reconstruction process. This *in situ* generated CoOOH differs from directly prepared CoOOH, as it provides more active sites and greater hydrophilicity for OER.^63^ The Raman peak located at 895 cm^−1^ corresponds to the characteristic vibration of Mo–O, which is associated with the MoO_4_^2−^ species.^64^ As the applied voltage increases, the peak intensity exhibits no significant change, indicating that while MoO_4_^2−^ is not the primary active site for the OER, it remains stably present on the electrocatalyst surface. This observation further demonstrates the outstanding stability of the B_4_O_5_(OH)_4_^2−^–CoFe-LDH/SO_4_^2−^–CoMoO_4_ nanohybrid. In the contour plot of the *in situ* Raman spectra for SO_4_^2−^–CoMoO_4_ (Fig. 5c), the distinct MoO_4_^2−^ peak is observed, while the relative intensity of the Co^3+^ peak appears weaker. As discussed earlier in the XPS analysis of Co 2p, the incorporation of Fe facilitates the formation of the active species Co^3+^, indicating that the construction of the amorphous/crystalline B_4_O_5_(OH)_4_^2−^–CoFe-LDH/SO_4_^2−^–CoMoO_4_ interface is more favorable for the generation of active species.

In the *in situ* ATR-IR spectra, four distinct absorption peaks at 1026, 1054, 1641, and 3267 cm^−1^ are attributed to OO_ad_, OOH_ad_, O_ad_, and OH_ad_ intermediates in the B_4_O_5_(OH)_4_^2−^–CoFe-LDH/SO_4_^2−^–CoMoO_4_ nanohybrid, respectively (Fig. S24).^65,66^ As the applied potential increases, the OH_ad_ signal not only becomes more intense but also exhibits a noticeable shift to higher wavenumbers, indicating alterations in the surrounding environment.^67^ Moreover, the appearance of the OOH_ad_ characteristic peak also corroborates the generation of CoOOH species during the surface reconstruction process, supporting the findings from *in situ* Raman. After the OER test, XPS measurements were conducted (Fig. S25). After the reaction, the peaks of Co 2p in the XPS spectra shift to higher binding energies, indicating the oxidation of Co^2+^ to CoOOH during the OER process.^68^ In contrast, the binding energy of Mo 3d remains unchanged after the OER test, while S and B elements persist on the electrocatalyst surface. This further indicates that SO_4_^2−^ and B_4_O_5_(OH)_4_^2−^ groups can stably exist during the OER process, providing sustained dual protection against Cl^−^ corrosion and facilitating the long-term stability of the electrocatalyst, while the CoOOH species acts as an active species in the OER process.

To further elucidate the electrocatalyst reaction mechanism, we further constructed structural models of B_4_O_5_(OH)_4_^2−^–CoFe-LDH/SO_4_^2−^–CoMoO_4_ and B_4_O_5_(OH)_4_^2−^–CoFe-LDH based on the above discussion and conducted density functional theory (DFT) calculations. Fig. 5d shows the adsorption structures of the OER intermediates (*OH, *O, and *OOH) on B_4_O_5_(OH)_4_^2−^–CoFe-LDH/SO_4_^2−^–CoMoO_4_, and the corresponding Gibbs free energies (Δ*G*) of the fundamental steps were calculated. As shown in Fig. 5e, the rate-determining steps (RDSs) for B_4_O_5_(OH)_4_^2−^–CoFe-LDH/SO_4_^2−^–CoMoO_4_ and B_4_O_5_(OH)_4_^2−^–CoFe-LDH are *O → *OOH (Δ*G*_3_) and *OOH → O_2_ (Δ*G*_4_), respectively. The Δ*G* value for B_4_O_5_(OH)_4_^2−^–CoFe-LDH/SO_4_^2−^–CoMoO_4_ is 1.67 eV, which is lower than that of B_4_O_5_(OH)_4_^2−^–CoFe-LDH (1.81 eV). Coupled with XPS analysis, it was found that the incorporation of SO_4_^2−^–CoMoO_4_ can modulate the electronic environment around B_4_O_5_(OH)_4_^2−^–CoFe-LDH, thereby optimizing the adsorption and desorption capabilities of the intermediate at the active sites, leading to a reduced energy barrier and accelerating the reaction kinetics.

Additionally, the Δ*G* for the key steps of the CER were calculated under the same conditions, with the RDS being *O + Cl^−^ (aq) → * + ClO^−^ (aq) (Fig. 5f). The Δ*G* values for B_4_O_5_(OH)_4_^2−^–CoFe-LDH/SO_4_^2−^–CoMoO_4_ and B_4_O_5_(OH)_4_^2−^–CoFe-LDH are 2.58 eV and 2.64 eV, respectively, both higher than the reaction barriers for the OER, indicating that the adsorption of Cl^−^ and desorption of ClO^−^ are more challenging at the reaction sites. The excellent corrosion resistance of B_4_O_5_(OH)_4_^2−^–CoFe-LDH/SO_4_^2−^–CoMoO_4_ and B_4_O_5_(OH)_4_^2−^–CoFe-LDH is attributed to the protection provided by the B_4_O_5_(OH)_4_^2−^/SO_*x*_^2−^ groups. Next, the resistance of B_4_O_5_(OH)_4_^2−^–CoFe-LDH/SO_4_^2−^–CoMoO_4_ and B_4_O_5_(OH)_4_^2−^–CoFe-LDH to Cl^−^ is compared (Fig. 5g). As described by the Arrhenius equation, the reaction rate is strongly dependent on the activation energy (*E*_a_). The reaction rate ratio of OER to CER follows an exponential relationship with −(*E*^OER^_a_–*E*^CER^_a_). By approximating the activation energy using the Gibbs free energy barriers ΔG_*OOH_ and Δ*G*_ClO^−^_ of the RDS for OER and CER, we evaluated the energy barrier differences for B_4_O_5_(OH)_4_^2−^–CoFe-LDH/SO_4_^2−^–CoMoO_4_ and B_4_O_5_(OH)_4_^2−^–CoFe-LDH. Notably, B_4_O_5_(OH)_4_^2−^–CoFe-LDH/SO_4_^2−^–CoMoO_4_ exhibits a more negative barrier difference (−0.91 eV), indicating a stronger preference for OER over CER, thereby demonstrating superior resistance to CER. This further indicates that sulfidation and heterointerface construction enhance the electrocatalyst's resistance to Cl^−^ corrosion not only by modulating the electronic structure for optimized reactant adsorption but also by providing a robust dual-anion protective layer, thereby enhancing both activity and long-term stability during seawater splitting.

**Fig. 6:**
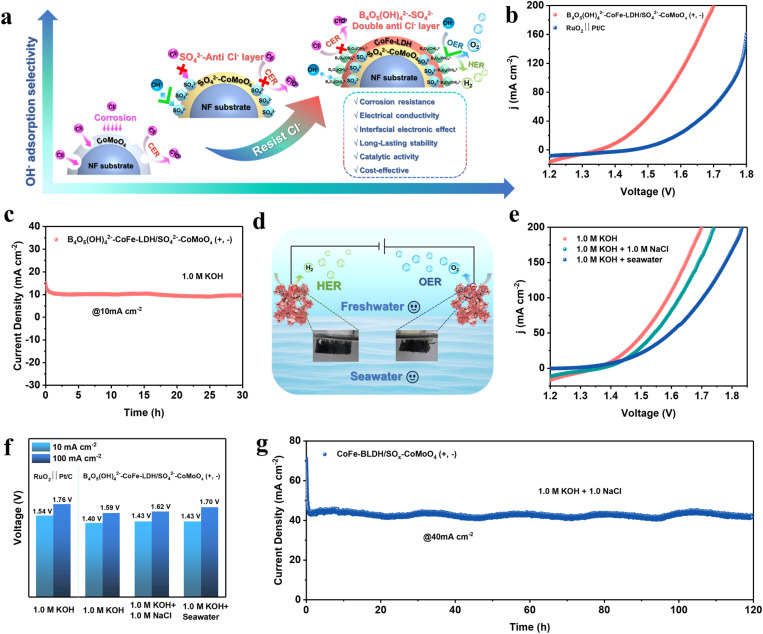
(a) Schematic illustration of enhanced Cl^−^ corrosion resistance at the B_4_O_5_(OH)_4_^2−^–CoFe-LDH/SO_4_^2−^–CoMoO_4_ interface in alkaline seawater. (b) LSV curves of the electrolyzer in 1.0 M KOH solution. (c) *i*–*t* curve in 1.0 M KOH solution. (d) Schematic representation of the overall water splitting process. (e) LSV curves of the electrolyzer in different electrolytes. (f) Voltages at different current densities and (g) *i*–*t* curve in 1.0 M KOH + 1.0 M NaCl solution.

The improved OH^−^ adsorption and Cl^−^ corrosion resistance of the B_4_O_5_(OH)_4_^2−^–CoFe-LDH/SO_4_^2−^–CoMoO_4_ nanohybrid can be attributed to three synergistic effects: (1) electrostatic repulsion from B_4_O_5_(OH)_4_^2−^/SO_4_^2−^ hinders Cl^−^ intrusion while promoting OH^−^ enrichment near the active sites; (2) electronic structure modulation by SO_4_^2−^–CoMoO_4_ lowers the reaction energy barrier; (3) Fe incorporation enhances *in situ* surface reconstruction, generating active CoOOH species and accelerating water dissociation (Fig. 6a).

Benefiting from its excellent activity and durability in the HER/OER, the B_4_O_5_(OH)_4_^2−^–CoFe-LDH/SO_4_^2−^–CoMoO_4_ nanohybrid is evaluated as a bifunctional electrocatalyst for overall water/seawater splitting. As shown in Fig. 6b, B_4_O_5_(OH)_4_^2−^–CoFe-LDH/SO_4_^2−^–CoMoO_4_ (+, −) shows a remarkably lower voltage for driving overall water splitting compared to commercial RuO_2_‖Pt/C, achieving 10 mA cm^−2^ at a voltage of only 1.4 V, which is significantly lower than the 1.54 V required by RuO_2_‖Pt/C. The B_4_O_5_(OH)_4_^2−^–CoFe-LDH/SO_4_^2−^–CoMoO_4_ nanohybrid (+, −) maintains stability in 1.0 M KOH for 30 h (Fig. 6c). Given the excellent anti-corrosion performance of the B_4_O_5_(OH)_4_^2−^–CoFe-LDH/SO_4_^2−^–CoMoO_4_ nanohybrid in seawater, its overall seawater splitting capability was further evaluated by comparing its performance in simulated alkaline seawater (1.0 M NaCl +1.0 M KOH) and natural alkaline seawater (seawater + 1.0 M KOH). In simulated alkaline seawater, B_4_O_5_(OH)_4_^2−^–CoFe-LDH/SO_4_^2−^–CoMoO_4_ (+, −) requires only an additional 0.03 V to overcome the interference from Cl^−^ in the electrolyte at 100 mA cm^−2^ (Fig. 6d–f), demonstrating its outstanding corrosion resistance. However, in natural seawater, B_4_O_5_(OH)_4_^2−^–CoFe-LDH/SO_4_^2−^–CoMoO_4_ (+, −) exhibits slightly reduced corrosion resistance, likely due to the presence of insoluble substances and microorganisms in natural seawater, which can be mitigated through pre-filtration.^8^ The overall seawater splitting stability of the B_4_O_5_(OH)_4_^2−^–CoFe-LDH/SO_4_^2−^–CoMoO_4_ nanohybrid was further assessed in simulated alkaline seawater at 60 °C. As shown in Fig. S26, the nanohybrid maintained stable electrolysis at a current density of 200 mA cm^−2^ for 80 hours, demonstrating excellent durability under industrially relevant thermal conditions. This performance of the B_4_O_5_(OH)_4_^2−^–CoFe-LDH/SO_4_^2−^–CoMoO_4_ nanohybrid surpasses that of most recently reported advanced electrocatalysts (Table S3), making it a promising bifunctional electrocatalyst for seawater splitting. Additionally, B_4_O_5_(OH)_4_^2−^–CoFe-LDH/SO_4_^2−^–CoMoO_4_ (+, −) maintains continuous electrolysis for 120 h in 1.0 M NaCl +1.0 M KOH (Fig. 6g), again indicating its excellent durability. Overall, the integration of dual-anion protection and amorphous/crystalline interface engineering endows the B_4_O_5_(OH)_4_^2−^–CoFe-LDH/SO_4_^2−^–CoMoO_4_ nanohybrid with outstanding bifunctional activity and long-term durability, establishing it as a benchmark candidate for practical seawater electrolysis.

## Conclusions

In summary, we successfully synthesized a B_4_O_5_(OH)_4_^2−^–CoFe-LDH/SO_4_^2−^–CoMoO_4_ nanohybrid that exhibits high activity and remarkable stability in both freshwater and seawater-buffered KOH solutions. The excellent performance could be attributed to the rational design of the amorphous/crystalline interface and the construction of B_4_O_5_(OH)_4_^2−^/SO_4_^2−^ dual-anion passivation layers, which modulate the electronic structures, accelerate interfacial electron transfer, increase the accessible active sites, and improve Cl^−^ resistance. Indeed, the B_4_O_5_(OH)_4_^2−^–CoFe-LDH/SO_4_^2−^–CoMoO_4_ nanohybrid maintains stable electrolysis for 720 hours in alkaline seawater at a current density of 1 A cm^−2^. The *in situ* Raman spectroscopy, *in situ* ATR-IR, and XPS results revealed that Fe atoms facilitated the *in situ* formation of CoOOH active species, which contributed to the enhanced OER activity. DFT calculations indicated that the incorporation of SO_4_^2−^–CoMoO_4_ could optimize the intermediate adsorption energies and improve the resistance to Cl^−^ corrosion, thereby increasing the activity and stability. This study provides valuable insights into the rational design of durable and high-performance electrocatalysts for seawater splitting *via* a combination of anion doping, phase engineering, and hetero-interface engineering.

## Author contributions

Tianqi Gao: data curation, methodology, investigation, writing – original draft. Wenzhe Wang: investigation. Zenong Zhang: writing – review and editing. Wanyu Li: investigation, data curation. Huanhuan Gao: investigation. Jiawei Liu: writing – review and editing. Xiaojun Zhao: writing – review and editing, methodology. Zhihong Liu: supervision, resources, writing – review and editing, funding acquisition. Yu Chen: resources, writing – review and editing, funding acquisition.

## Conflicts of interest

The authors declare no conflicts of interest.

## Supplementary Material

SC-OLF-D5SC03775A-s001

## Data Availability

The data supporting this article have been included as part of the SI. Supplementary information is available. See DOI: https://doi.org/10.1039/d5sc03775a.
