# Supplementary material for: Dual-anion regulation engineering enhances chloridion corrosion resistance for long-lasting industrial-scale seawater splitting
Source: Chem Sci. 2025 Jul 29;16(34):15684–96. doi: 10.1039/d5sc03775a (PMC12322833; doi:10.1039/d5sc03775a)
Supplement: SC-016-D5SC03775A-s001 [file SC-016-D5SC03775A-s001.pdf]

Supplementary Information (SI) for Chemical Science.
This journal is © The Royal Society of Chemistry 2025

Electronic supplementary information

Dual-anion regulation engineering enhances the chloridion corrosion resistance for long-lasting industrial-scale seawater splitting

Tianqi Gao,[a] *Wenzhe Wang,*[a] *Zenong Zhang,*[a] *Wanyu Li,*[a] *Huanhuan Gao,*[a] *Jiawei Liu,**[b] *Xiaojun Zhao,**[c] *Zhihong Liu,**[a] *and Yu Chen**[d]

[a] Key Laboratory for Macromolecular Science of Shaanxi Province, School of Chemistry and Chemical Engineering, Shaanxi Normal University, Xi'an 710062, P. R. China

[b] Department of Chemical and Biological Engineering, The Hong Kong University of Science and Technology, Clear Water Bay, Kowloon, Hong Kong 999077, P. R. China

[c] School of Metallurgical Engineering, Xi'an University of Architecture and Technology, Xi'an 710055, P. R. China

[d] School of Materials Science and Engineering, Shaanxi Normal University, Xi'an 710119, P. R. China

*Corresponding authors.
E-mail: jiaweiliu@ust.hk (J. Liu), xjzhao@xauat.edu.cn (X. Zhao), liuzh@snnu.edu.cn (Z. Liu), ndchenyu@gmail.com (Y. Chen).

Experimental Section

Synthesis of $CoMoO_4$ nanorods: Firstly, a patch of nickel foam (NF, 2.0 cm × 3.0 cm) was sequentially cleaned in acetone, HCl solution (6.0 M), deionized water, and ethanol for 25 min. Then, 3.5 mM $Co(NO_3)_2{\cdot}6H_2O$, 1.0 mM $(NH_4)_6Mo_7O_{24}{\cdot}4H_2O$, 5 mM urea, and 2.5 mM NH_4F were dissolved in 40 mL deionized water with stirring for 30 min. The solution and cleaned NF were transferred into a Teflon-lined stainless steel autoclave and preserved at 150 °C for 6 h. After naturally cooling to room temperature, the obtained precursor was washed with deionized water and dried overnight at 60 °C. Subsequent calcination in air at 400 °C for 2 h resulted in the formation of $CoMoO_4$ nanorods. The loading mass of $CoMoO_4$ on the NF was 36.5±0.5 mg cm^{-2}.

Synthesis of SO_4^{2-}-$CoMoO_4$ nanorods: SO_4^{2-}-$CoMoO_4$ nanorods were prepared by a typical hydrothermal method. Specifically, 0.3 g of thioacetamide was dissolved in 40 mL deionized water. The solution and $CoMoO_4$ on NF were then transferred into a Teflon-lined stainless steel autoclave and preserved at 120 °C for 1h, 3 h and 6 h. After naturally cooling to room temperature, the obtained SO_4^{2-}-$CoMoO_4$-1h, SO_4^{2-}-$CoMoO_4$-3h and SO_4^{2-}-$CoMoO_4$-6h were washed with deionized water and dried overnight at 60 °C. The loading mass of SO_4^{2-}-$CoMoO_4$ on the NF was 41.5±0.5 mg cm^{-2}.

Synthesis of $B_4O_5(OH)_4^{2-}$-CoFe-LDH/SO_4^{2-}-$CoMoO_4$ nanohybrid: To obtain $B_4O_5(OH)_4^{2-}$-CoFe-LDH/SO_4^{2-}-$CoMoO_4$ nanohybrid, the obtained SO_4^{2-}-$CoMoO_4$ nanorods precursor was placed in a mixed solution (3.5 mM $Co(NO_3)_2{\cdot}6H_2O$, 1.16 mM $Fe(NO_3)_3{\cdot}9H_2O$, and 10 mM $NH_4B_5O_8{\cdot}4H_2O$ dissolved in 40 ml deionized water), stirred for 2 h, 3 h and 6 h at 25 °C. Then, the products were washed with deionized water and dried overnight at 60 °C. The $B_4O_5(OH)_4^{2-}$-CoFe-LDH-2h/SO_4^{2-}-$CoMoO_4$, $B_4O_5(OH)_4^{2-}$-CoFe-LDH-3h/SO_4^{2-}-$CoMoO_4$ and $B_4O_5(OH)_4^{2-}$-CoFe-LDH-6h/SO_4^{2-}-$CoMoO_4$/NF were obtained. The loading mass of $B_4O_5(OH)_4^{2-}$-CoFe-LDH/SO_4^{2-}-$CoMoO_4$ on the NF was 38±0.5 mg cm^{-2}.

For comparison, crystalline/crystalline $B_4O_5(OH)_4^{2-}$-CoFe-LDH/SO_4^{2-}-$CoMoO_4$ nanohybrid was prepared by a typical hydrothermal method. The obtained SO_4^{2-}-$CoMoO_4$ nanorods precursor and the mixed solution (3.5 mM $Co(NO_3)_2{\cdot}6H_2O$, 1.16 mM $Fe(NO_3)_3{\cdot}9H_2O$ and 10 mM $NH_4B_5O_8{\cdot}4H_2O$ dissolved in 40 ml deionized water) were then transferred into a Teflon-lined stainless steel autoclave and preserved at 120 °C for 12 h. After cooling down to room temperature, the obtained crystalline/crystalline $B_4O_5(OH)_4^{2-}$-CoFe-LDH/SO_4^{2-}-$CoMoO_4$ nanohybrid was washed with deionized water and dried overnight at 60 °C.

For simplicity, the supporting material NF is omitted in the main text.

Materials characterization: The morphology and microstructure of the as-prepared samples were characterized by field emission scanning electron microscopy (FESEM, SU8220, 20 kV) and transmission electron microscope (TEM, FEI Tecnai F20). X-ray diffraction (XRD) was measured by Bruker D8 ADVANCE with Cu-Kα radiation. X-ray photoelectron spectroscopy (XPS) investigation was conducted on Axis Ultra DLD with Al Kα as a radiation exciting source (AXIS ULTRA). Wettability measurements were carried out with the Optical Contact Angle Measuring Device (KRUSS-DSA100). Fourier transform infrared (FTIR TENSOR27, Bruker, Germany) spectra were collected using the standard KBr disk method with the range of 400-4000 cm^{-1}. The color reaction of ClO^- was determined using a UV-vis spectrophotometer (UV-6100, Shanghai Mapada Instruments, China). *In-suit* Raman experiments were carried out with a confocal microscope Raman spectroscopy (LabRAM Odyssey France), and the excitation wavelength was 532 nm (100 mW). *In suit* Infrared Spectroscopy (IR) experiments were collected using a Bruker INVENIOS spectrometer.

Electrochemical measurements: All of the electrochemical measurements were conducted using an electrochemical workstation (CHI760E, CH Instruments, Shanghai, China). A graphite rod and Hg/HgO electrode were processed as the counter and reference electrode, respectively. All linear sweep voltammetry (LSV) tests were conducted with 90% iR compensation. As-

prepared samples were used as working electrodes (1 cm × 0.4 cm). LSV for both OER and HER was conducted at a scan rate of 5 mV s^{-1}. Cyclic voltammetry (CV) mode with various scan rates from 20 to 100 mV s^{-1} was measured in the non-Faradaic region between 0.624 and 0.724 V (vs RHE) for OER to evaluate the electrochemical double-layered capacitance (C_{dl}). Electrochemical impedance spectroscopy (EIS) measurements were collected within a frequency range of 0.01 Hz to 100 kHz. All potentials were converted to the potentials versus the reversible hydrogen electrode (RHE) according to the Nernst equation ($E_{RHE} = E_{Hg/HgO} + 0.059\ pH + 0.098V$).

Theoretical calculation: In density functional theory (DFT) calculations, structural optimizations were performed by Vienna *Ab-initio* Simulation Package (VASP)[1] with the projector augmented wave (PAW) method.[2] The exchange-functional was treated using the Perdew-Burke-Ernzerhof (PBE)[3] functional, in combination with the DFT-D3 correction.[4] The cut-off energy of the plane-wave basis was set at 450 eV in structural optimizations. For optimization of lattice size of CoFe-LDH (001) and geometry of $CoMoO_4$(110)-2S/CoFe-LDH (001), the Brillouin Zone integration was performed with a Monkhorst[5] *k*-point mesh of 2×1×1. The self-consistent calculations applied a convergence energy threshold of 10^{-5} eV. The equilibrium geometries and lattice constants were optimized with maximum stress on each atom within 0.02 eV $Å^{-1}$. Spin polarization method was adopted to describe magnetism of slab models. The Hubbard U correction[6,7] was added to describe 3d orbitals of Co atoms, 3d orbitals of Fe atoms, and 4d orbitals of Mo atoms, where $U_{Co} = 3.32$ eV, $U_{Fe} = 5.3$ eV, $U_{Mo} = 4.38$ eV.[8]

In Gibbs free energy calculation, we built the hydrogen adsorption model by employing the computational hydrogen electrode (CHE) model developed by Nørskov et al.[9]

Elementary steps of chlorine evolution reaction (CER) to ClO^- were described as:

$* + OH^-(aq) \rightarrow *OH + e^-$

$*OH + OH^-(aq) \rightarrow *O + H_2O(l) + e^-$

$*O + Cl^-(aq) \rightarrow * + ClO^-(aq)$

In reaction pathways, * represents the bare surface of slab model. *i represents slab model with intermediate i adsorbed on surface. Gibbs free energy of *i was calculated as $G = E + G(T) - 0.0592pH - eU$. E represents the total energy of *i. G(T) represents the thermal correction of *i. G(T) of *i was obtained by vaspkit interface, as same as G(T) of H_2, H_2O, Cl_2 in in their gas phase.[10] G(T) contains two terms of correction, including zero-point energy (ZPE), product of temperature and entropy (TS). The Kelvin temperature T was set at 298.15K. Gibbs free energy of OH^-, O_2, Cl^-, ClO^- in their aqueous phase, H_2O in its liquid phase were referenced to standard molar free energy of formation.[11] pH value was set at 14 to simulate basic medium for OER and CER. Besides, the applied potential U was set at 0 V and 0.4 V vs. RHE, respectively.

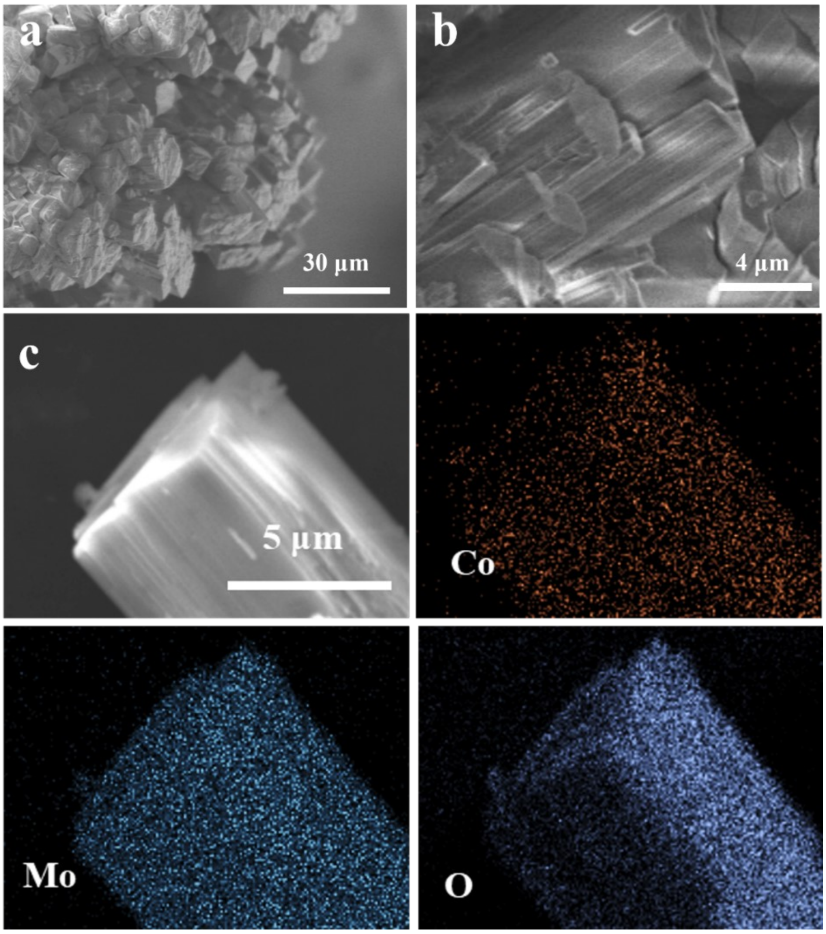

Fig. S1 (a, b) SEM and (c) elemental mapping images of $CoMoO_4$ on NF.

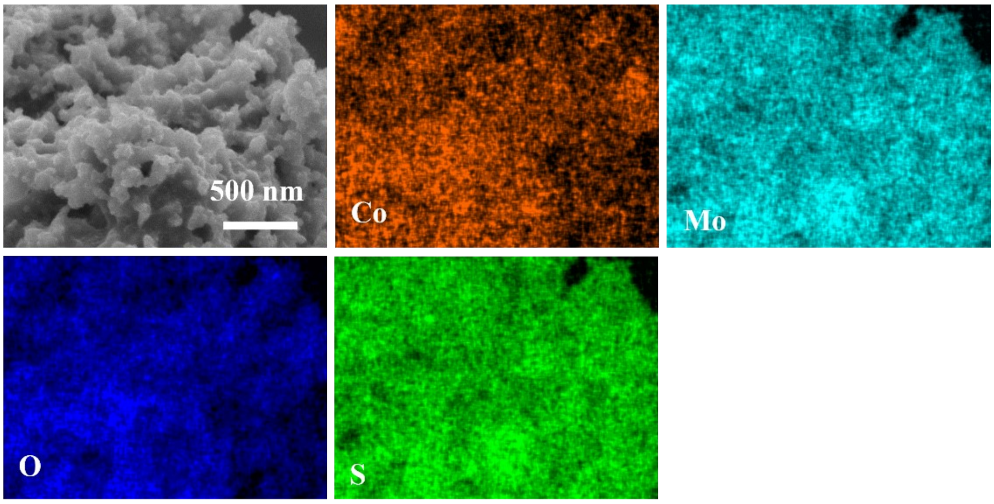

Fig. S2 Elemental mapping images of SO_4^{2-}-$CoMoO_4$ on NF.

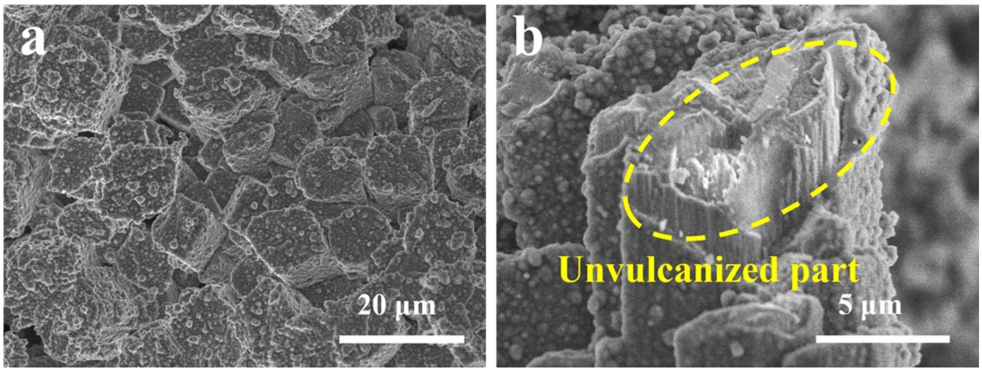

Fig. S3 SEM images of SO_4^{2-}-$CoMoO_4$-1h on NF.

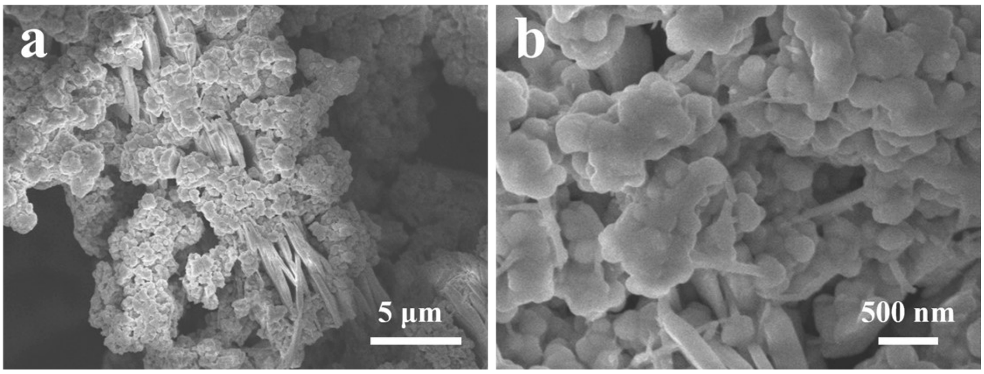

Fig. S4 SEM images of SO_4^{2-}-$CoMoO_4$-6h on NF.

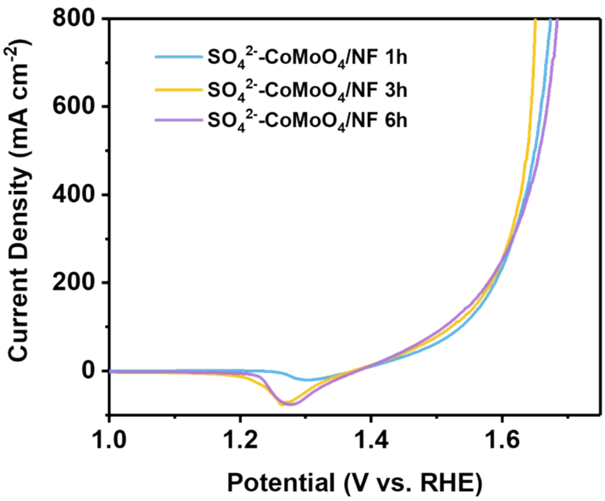

Fig. S5 LSV curves of SO_4^{2-}-$CoMoO_4$-1h, SO_4^{2-}-$CoMoO_4$-3h and SO_4^{2-}-$CoMoO_4$-6h in 1.0 M KOH solution.

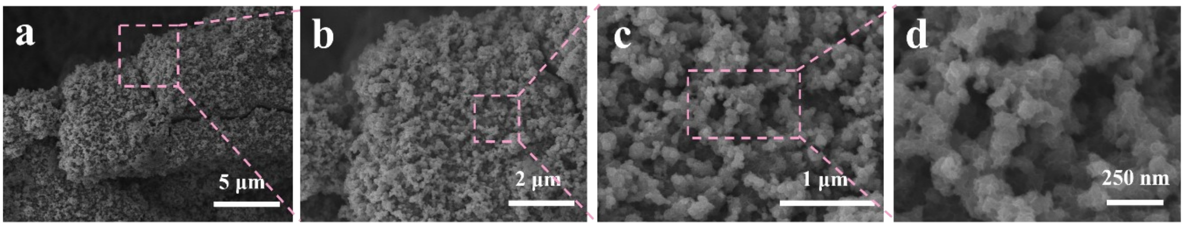

Fig. S6 SEM images of $B_4O_5(OH)_4^{2-}$-CoFe-LDH/SO_4^{2-}-$CoMoO_4$-6h on NF.

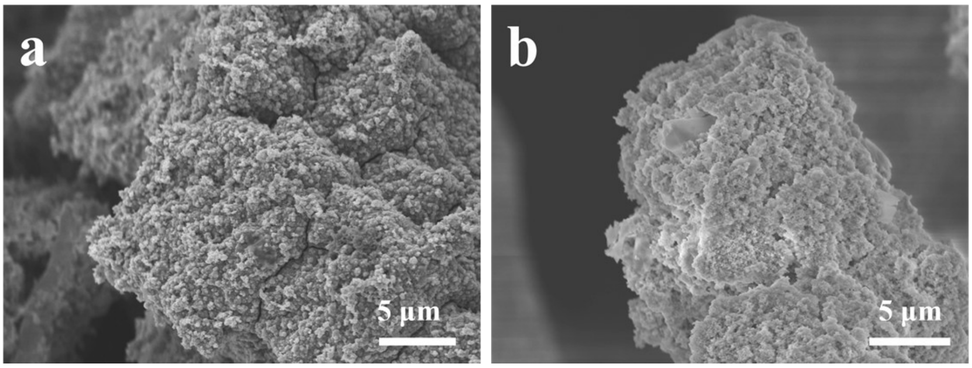

Fig. S7 SEM images of (a) $B_4O_5(OH)_4^{2-}$-CoFe-LDH-2h/SO_4^{2-}-$CoMoO_4$ and (b) $B_4O_5(OH)_4^{2-}$-CoFe-LDH-6h/SO_4^{2-}-$CoMoO_4$ on NF.

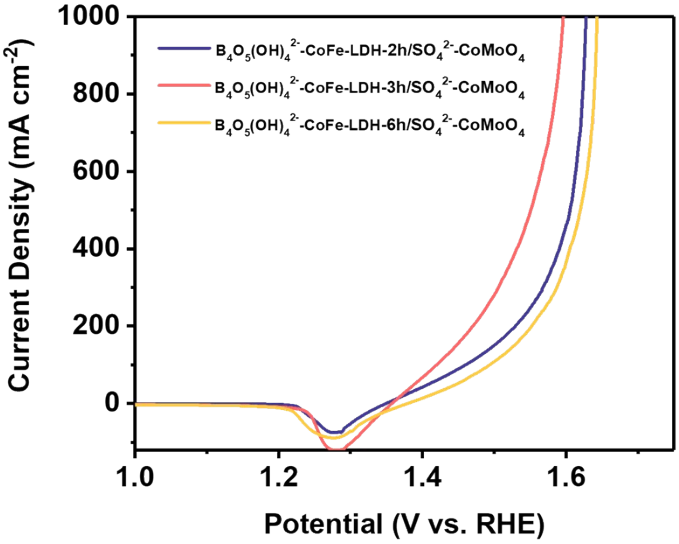

Fig. S8 LSV curves of $B_4O_5(OH)_4^{2-}$-CoFe-LDH-2h/SO_4^{2-}-$CoMoO_4$, $B_4O_5(OH)_4^{2-}$-CoFe-LDH-3h/SO_4^{2-}-$CoMoO_4$, and $B_4O_5(OH)_4^{2-}$-CoFe-LDH-6h/SO_4^{2-}-$CoMoO_4$ in 1.0 M KOH solution.

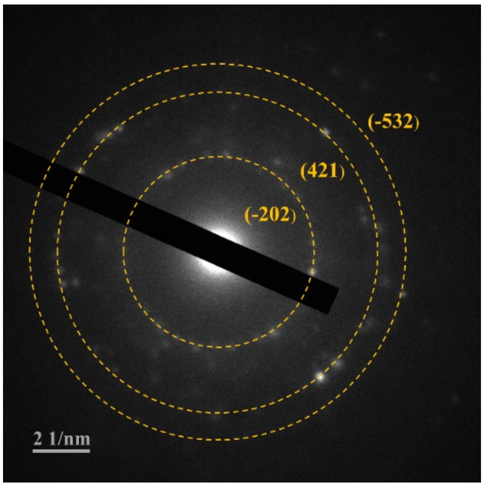

Fig. S9 SAED images of $B_4O_5(OH)_4^{2-}$-CoFe-LDH/SO_4^{2-}-$CoMoO_4$ nanohybrid.

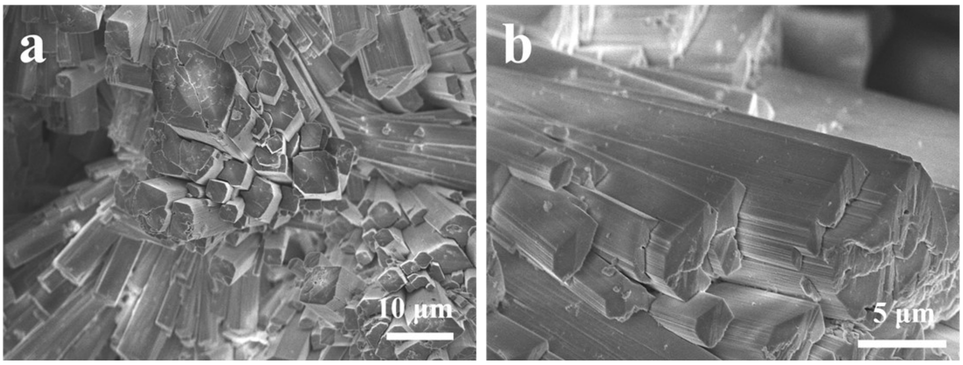

Fig. S10 SEM images of $B_4O_5(OH)_4^{2-}$-CoFe-LDH/$CoMoO_4$ on NF.

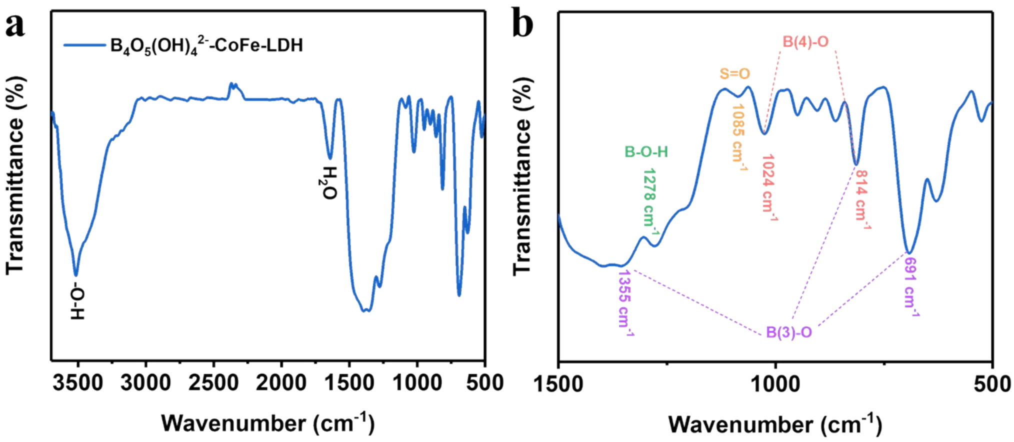

Fig. S11 (a, b) FTIR spectra of $B_4O_5(OH)_4^{2-}$-CoFe-LDH.

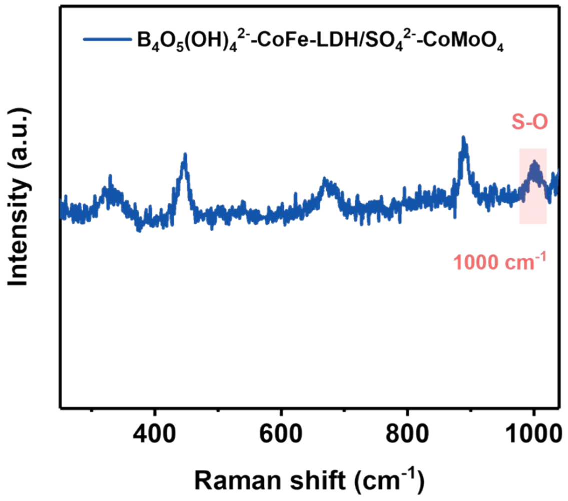

Fig. S12 Raman spectra of $B_4O_5(OH)_4^{2-}$-CoFe-LDH/SO_4^{2-}-$CoMoO_4$.

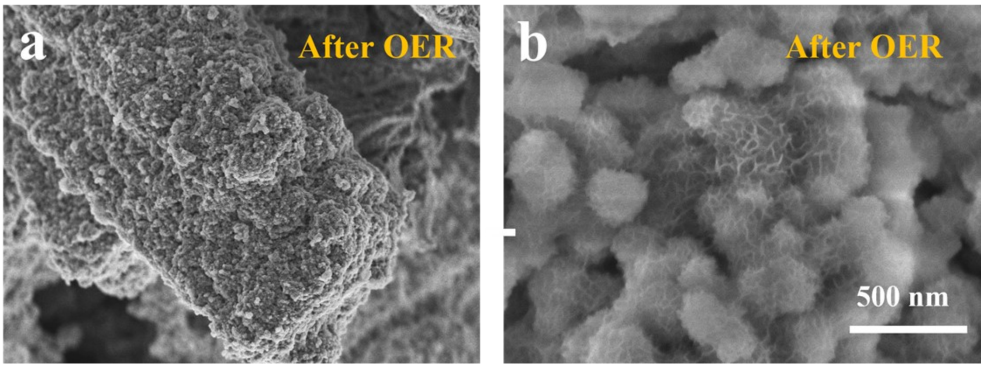

Fig. S13 SEM images of $B_4O_5(OH)_4^{2-}$-CoFe-LDH/SO_4^{2-}-$CoMoO_4$ after OER test.

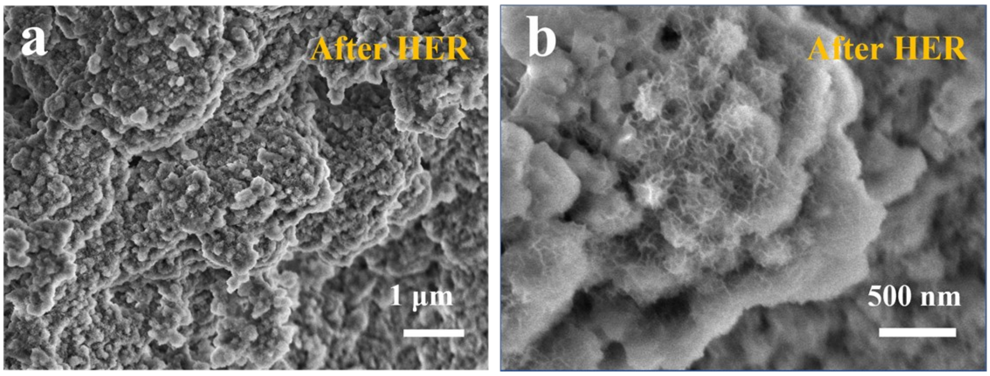

Fig. S14 SEM images of $B_4O_5(OH)_4^{2-}$-CoFe-LDH/SO_4^{2-}-$CoMoO_4$ after HER test.

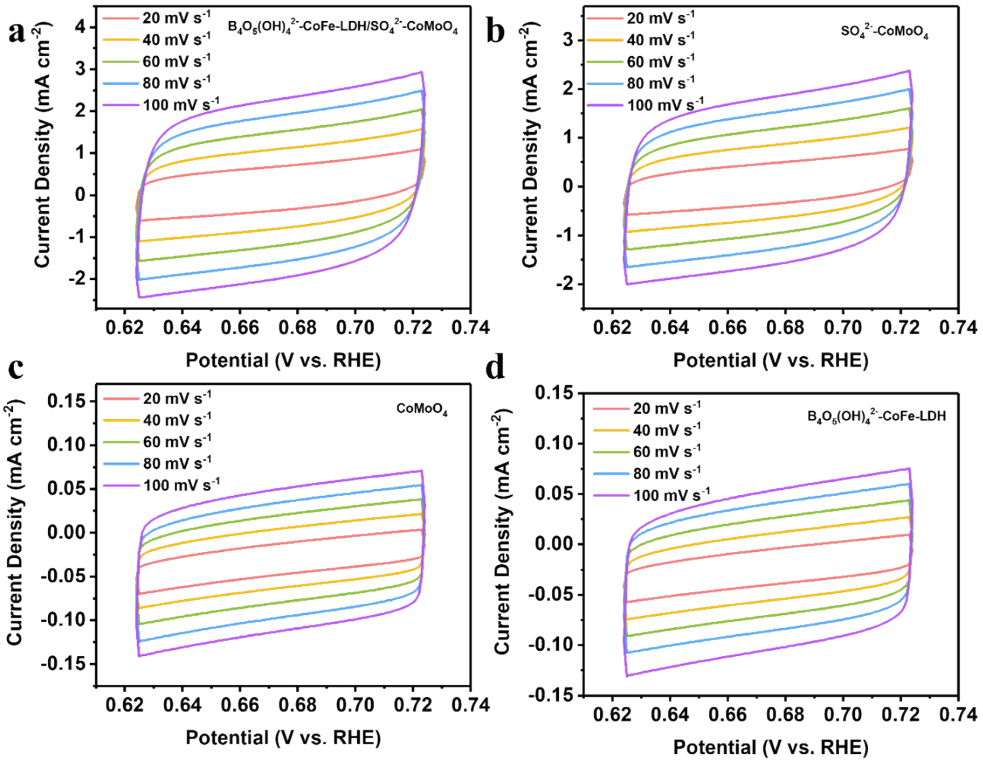

Fig. S15 The CV curves of (a) $B_4O_5(OH)_4^{2-}$-CoFe-LDH/SO_4^{2-}-$CoMoO_4$, (b) SO_4^{2-}-$CoMoO_4$, (c) $CoMoO_4$, and (d) $B_4O_5(OH)_4^{2-}$-CoFe-LDH for OER in 1.0 M KOH.

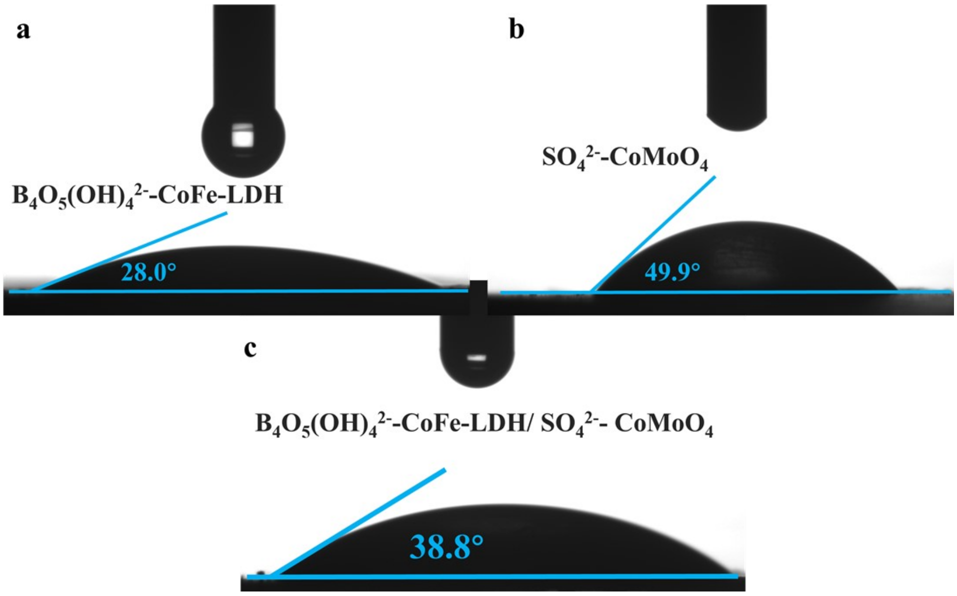

Fig. S16 Water contact angle on the surface of (a) $B_4O_5(OH)_4^{2-}$-CoFe-LDH, (b) SO_4^{2-}-$CoMoO_4$, and (c) $B_4O_5(OH)_4^{2-}$-CoFe-LDH/SO_4^{2-}-$CoMoO_4$.

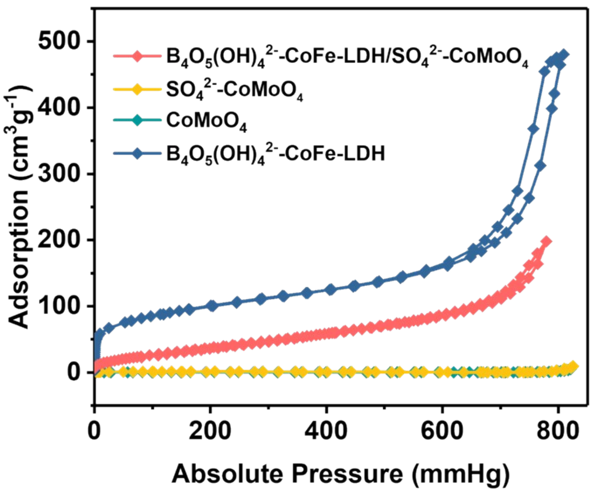

Fig. S17 N_2 adsorption-desorption isotherms.

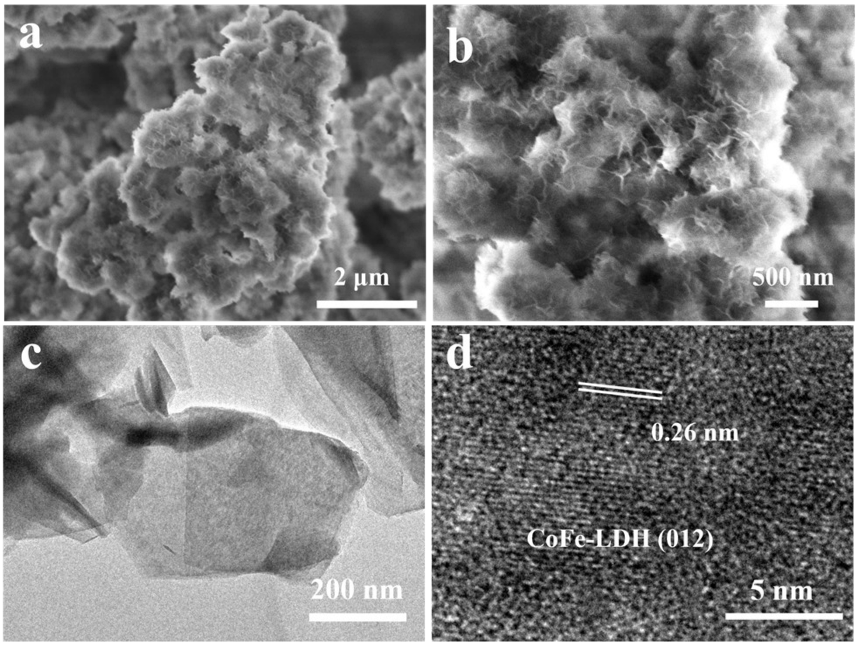

Fig. S18 (a, b) SEM, (c) TEM and (d) HR-TEM images of crystalline/crystalline $B_4O_5(OH)_4^{2-}$-CoFe-LDH/SO_4^{2-}-$CoMoO_4$. To clarify the role of the amorphous structure in enhancing electrocatalytic performance, we have synthesized a crystalline/crystalline counterpart of the $B_4O_5(OH)_4^{2-}$-CoFe-LDH/SO_4^{2-}-$CoMoO_4$ electrocatalyst. The crystalline/crystalline $B_4O_5(OH)_4^{2-}$-CoFe-LDH/SO_4^{2-}-$CoMoO_4$ nanohybrid was synthesized through a hydrothermal treatment. The SEM images consistently reveal that the SO_4^{2-}-$CoMoO_4$ nanorods are

encapsulated by $B_4O_5(OH)_4^{2-}$-CoFe-LDH nanosheets (Fig. S18a and b†). TEM and HRTEM images of the outer $B_4O_5(OH)_4^{2-}$-CoFe-LDH nanosheets reveal the 2D morphology with a lattice spacing of 0.26 nm, corresponding to the (012) crystalline plane of CoFe-LDH (Fig. S18c and d†), which confirms that well-crystallized crystalline/crystalline $B_4O_5(OH)_4^{2-}$-CoFe-LDH/SO_4^{2-}-$CoMoO_4$ are obtained.

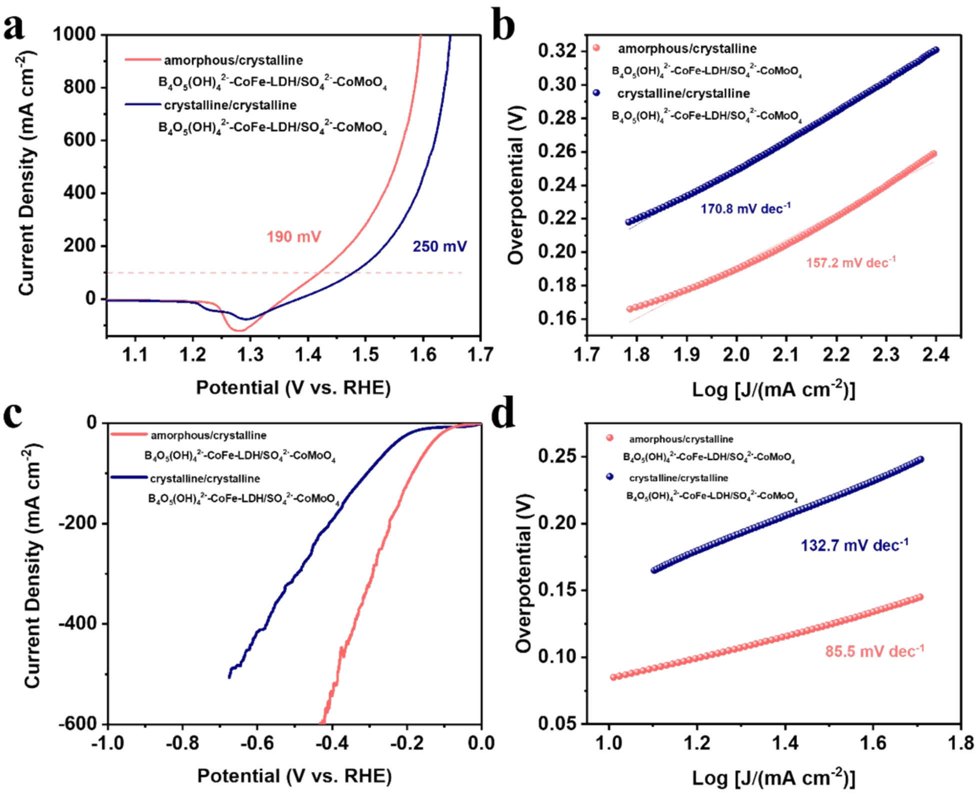

Fig. S19 (a, b) OER LSV curves and Tafel slopes; (c, d) HER LSV curves and Tafel slopes of amorphous/crystalline and crystalline/crystalline $B_4O_5(OH)_4^{2-}$-CoFe-LDH/SO_4^{2-}-$CoMoO_4$ in 1.0 M KOH solution.

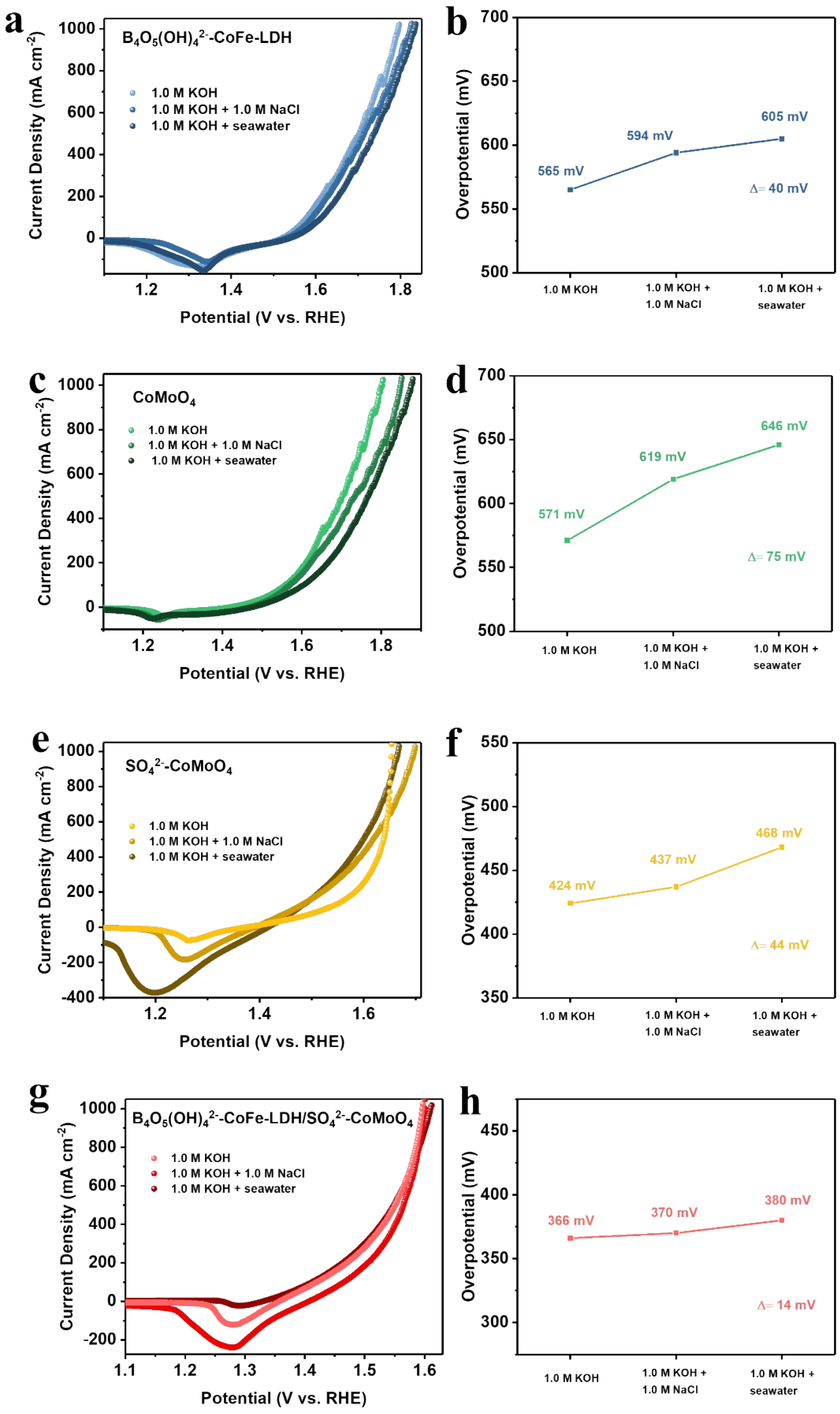

Fig. S20 LSV curves of (a) $B_4O_5(OH)_4^{2-}$-CoFe-LDH, (c) $CoMoO_4$, (e) SO_4^{2-}-$CoMoO_4$, and (f) $B_4O_5(OH)_4^{2-}$-CoFe-LDH/SO_4^{2-}-$CoMoO_4$ for OER in different electrolytes. (b, d, f, h) Corresponding overpotential under different electrolytes.

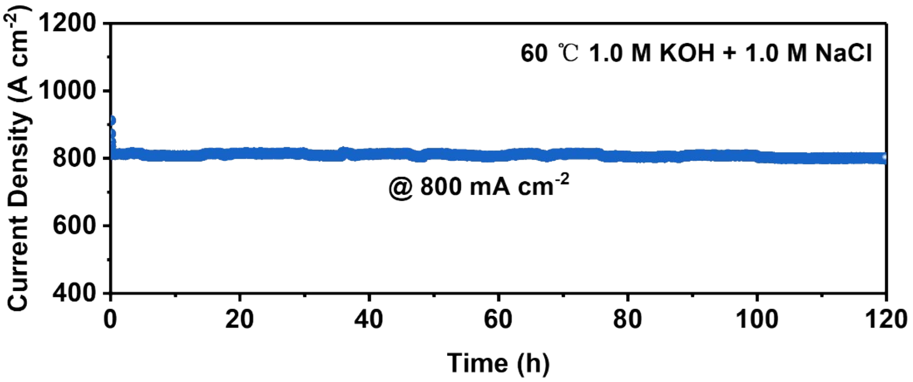

Fig. S21 *i*-t curve for OER in 1.0 M NaCl + 1.0 M KOH solution at 60 °C.

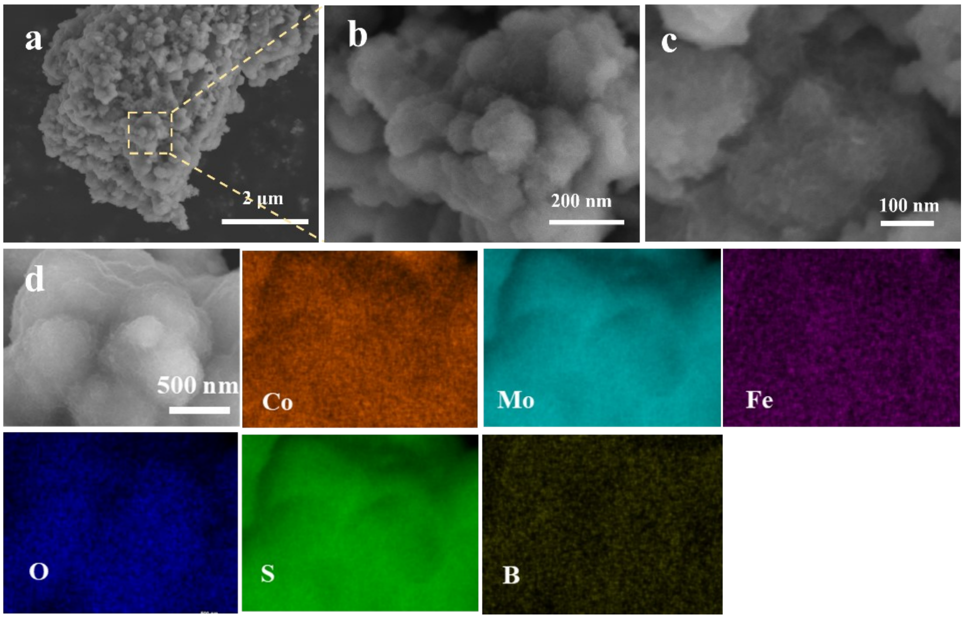

Fig. S22 (a-c) SEM and (d) elemental mapping images of $B_4O_5(OH)_4^{2-}$-CoFe-LDH/SO_4^{2-}-$CoMoO_4$ after the durability test at 1 A cm^{-2}.

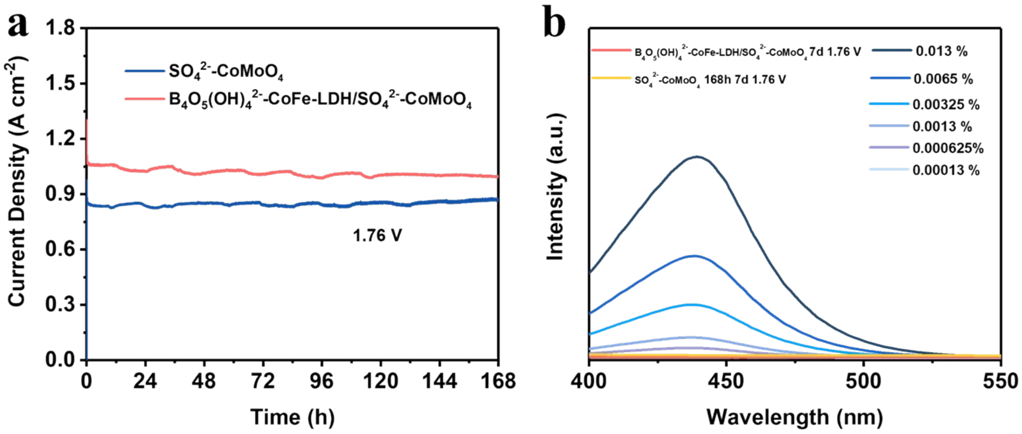

Fig. S23 (a) Durability test for $B_4O_5(OH)_4^{2-}$-CoFe-LDH/SO_4^{2-}-$CoMoO_4$ and SO_4^{2-}-$CoMoO_4$ at 1.76 V. (b) UV- vis spectra of ClO^- formation in 1.0 M KOH + 1.0 M NaCl after the OER stability tests.

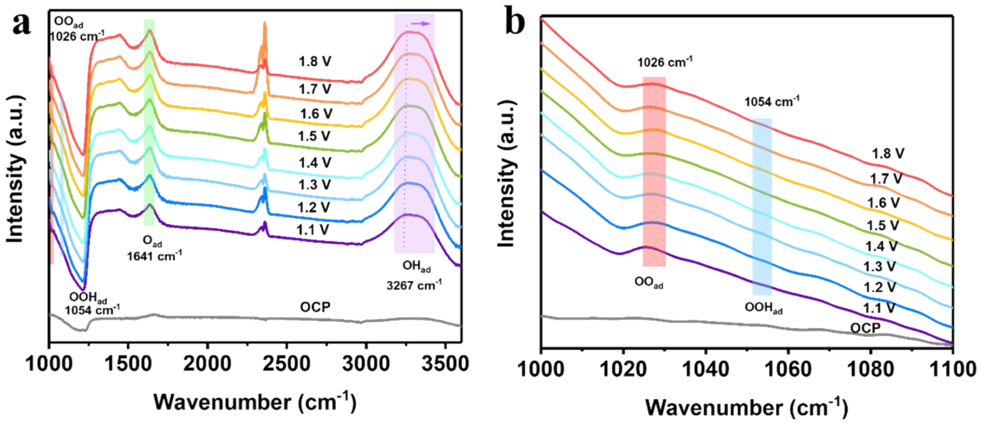

Fig. S24 (a, b) *In situ* ATR-IR spectra of $B_4O_5(OH)_4^{2-}$-CoFe-LDH/SO_4^{2-}-$CoMoO_4$.

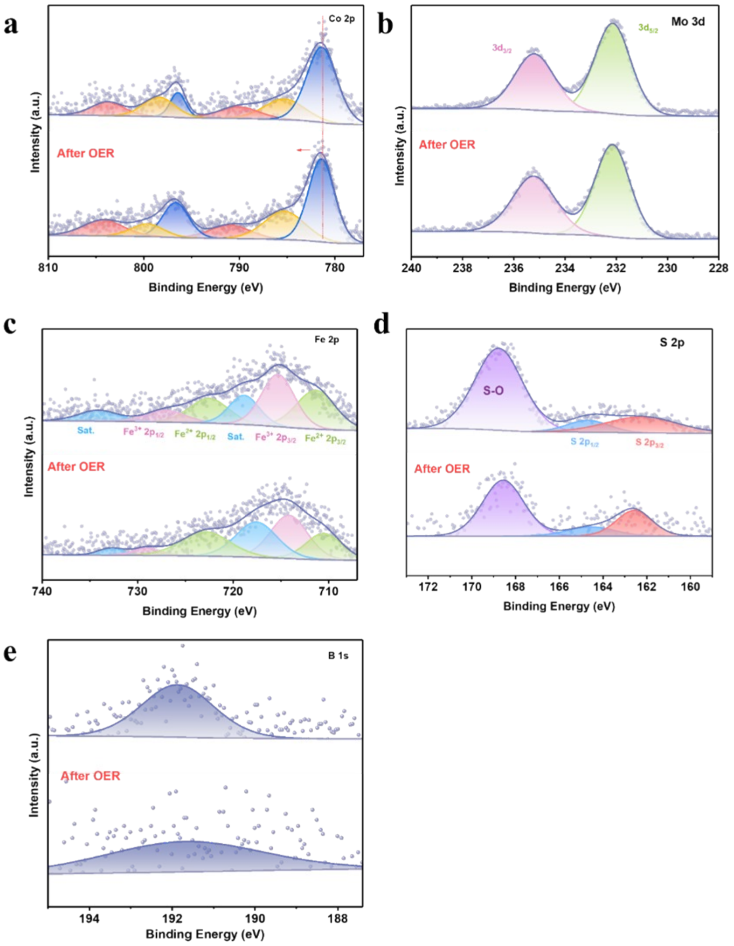

Fig. S25 XPS spectra of $B_4O_5(OH)_4^{2-}$-CoFe-LDH/SO_4^{2-}-$CoMoO_4$ after OER test in 1.0 M KOH solution.

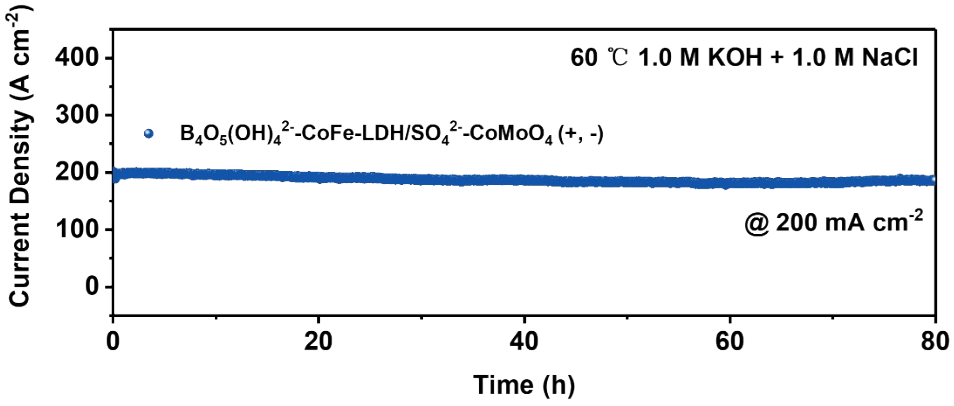

Fig. S26 *i*-t curve for overall water splitting in 1.0 M NaCl + 1.0 M KOH solution at 60 °C.

Tabel S1. OER performance of different catalysts in 1.0 M KOH solution.

Catalysts	J (mA cm^{-2})	η (mV)	Reference
$B_4O_5(OH)_4^{2-}$-CoFe-LDH/SO_4^{2-}-$CoMoO_4$	100	190	This work
$Fe_4N/Co_3N/MoO_2$	100	222	Adv. Mater. **2024**, 36, 2405852
$NiMoO_4$@$Mo_{15}Se_{19}/NiSe_2$	100	195	Adv. Energy Mater. **2024**, 14, 2304546
V_O-$CoMoVO_x$	10	248	Adv. Funct. Mater. **2025**, 2425503
$FeNi(OH)_x$-NiS@$Ni(OH)_2$	10	254.9	Adv. Funct. Mater. **2024**, 34, 2409849
Fe-P-CMO	10	238	Appl. Catal. B: Environ. **2024**, 346, 123741
VFe-NiS	10	190	Appl. Catal. B: Environ. **2024**, 359, 124461
Mo-Ni_2P/Fe_xP-V/NFF	100	246	Adv. Funct. Mater. **2024**, 34, 2400397
Ni-$NiMoO_4$/NF	100	298	Chem. Eng. J. **2024**, 484, 149498
Vo-$CoMoO_4$@Cu_2S/CF	10	181	Chem. Eng. J. **2023**, 477, 147016

Tabel S2. HER performance of different catalysts in 1 M KOH solution.

Catalysts	J (mA cm^{-2})	η (mV)	Reference
$B_4O_5(OH)_4^{2-}$-CoFe-LDH/SO_4^{2-}-$CoMoO_4$	10	85	This work
$CoPt_3$/FCWO-NS	10	135	Appl. Catal. B: Environ. **2024**, 342, 123387
NF/NiSe/Ni_3Se_2-Fe	10	144	Chem. Eng. J. **2024**, 488, 150996
Pd-$MoS_x(OH)_y$	10	93	Chem. Eng. J. **2024**, 497, 154524
Fe/W-Ni_3S_2	10	174	Small **2024**, 20, 2311770
$NiFeSe_4$/$NiSe_2$	10	121	J. Mater. Chem. A, **2024**, 12, 1714
V-NiS/NiS_2	10	93	Adv. Energy Mater. **2023**, 13, 2300978
FeMo@CoNi-OH/Ni_3S_2	10	89	Chem. Eng. J. **2023**, 468, 143605
NiV-LDH@Mn_2O_3	50	185	J. Mater. Chem. A, **2024**, 12, 21385

Tabel S3. Overall water-splitting performance of different catalysts in 1.0 M KOH solution.

Catalysts	E (V) @ 10 mA cm^{-2}	Reference
$B_4O_5(OH)_4^{2-}$-CoFe-LDH/SO_4^{2-}-$CoMoO_4$	1.40	This work
HPS-NiMo	1.45	ACS Nano **2024**, 18, 16312
R-$Ni_{0.6}Fe_{0.4}OOH$	1.52	Adv. Sci. **2024**, 11, 2408754
$FeNi(OH)_x$-NiS@$Ni(OH)_2$	1.57	Adv. Funct. Mater. **2024**, 34, 2409849
$FeNi_2S_4$	1.63	Small **2024**, 20, 2311627
VFe-NiS	1.48	Appl. Catal. B: Environ. **2024**, 359, 124461
Mo-Ni_2P/Fe_xP-V/NFF	1.49	Adv. Funct. Mater. **2024**, 34, 2400397
NiO/NiCoP	1.56	J. Mater. Chem. A, **2024**, 12, 18313
Vo-$CoMoO_4$@Cu_2S/CF	1.47	Chem. Eng. J. **2023**, 477, 147016

References

1 J. Hafner, *J. Comput. Chem.*, 2008, **29**, 2044-2078.

2 P. E. Blöchl, *Phys. Rev. B*, 1994, **50**, 17953.

3 J. P. Perdew, K. Burke and M. Ernzerhof, *Phys. Rev. Lett.*, 1996, **77**, 3865.

4 S. Grimme, *J. Comput. Chem.*, 2006, **27**, 1787.

5 H. J. Monkhorst and J. D. Pack, *Phys. Rev. B*, 1976, **13**, 5188.

6 V. I Anisimov, J. Zaanen and O. K. Andersen, *Phys. Rev .B*, 1991, **44**, 943.

7 V. I Anisimov I, F. Aryasetiawan and A. I Lichtenstein, *J. Phys. Condens. Matter*, 1997, **9**, 767.

8 J. M. Munro, K. Latimer, M. K. Horton, S. Dwaraknath and K. A. Persson, *Npj Comput. Mater.*, 2020, **6**, 112.

9 E. Skúlason, G. S. Karlberg, J. Rossmeisl, T. Bligaard, J. Greeley, H. Jónsson and J. K. Nørskov, *Phys. Chem.*, 2007, **9**, 3241-3250.

10 V. Wang, N. Xu, J.-C. Liu, G. Tang and W.-T. Geng, *Comput. Phys. Commun.*, 2021, **267**, 108033.

11 CRC Handbook of Chemistry and Physics, 97th Edition, 2016.
